# Supplementary material for: Non-pharmaceutical interventions in complementary and alternative medicine for insomnia in breast cancer survivors: a protocol for a systematic review and network meta-analysis
Source: BMJ Open. 2023 May 4;13(5):e071784. doi: 10.1136/bmjopen-2023-071784 (PMC10163449; doi:10.1136/bmjopen-2023-071784)
Supplement: Supplementary data [file bmjopen-2023-071784supp001.pdf]

**Search strategies**

**Pubmed:**

(((("breast neoplasms"[Mesh]) OR (breast cancer[tiab]) OR (breast tumor[tiab]) OR (mammary cancer[tiab]) OR (breast carcinoma[tiab])) AND (("sleep initiation and maintenance disorders"[Mesh]) OR (insomnia[tiab]))) AND ((("complementary therapies"[Mesh]) OR (complementary medicine[tiab]) OR (alternative medicine[tiab]) OR (alternative therapies[tiab]) OR (integrative medicine[tiab])) OR (("acupuncture"[Mesh]) OR (electroacupuncture[tiab]) OR (auricular acupuncture[tiab])) OR ("cognitive behavioral therapy"[Mesh]) OR ("massage"[Mesh]) OR (tuina[tiab]) OR ("mindfulness"[Mesh]) OR ("meditation"[Mesh]) OR ("mind-body therapies"[Mesh]) OR ("yoga"[Mesh]) OR (("tai ji"[Mesh]) OR (tai chi[tiab])) OR ("qigong"[Mesh]) OR (("exercise"[Mesh]) OR (physical exercise[tiab]) OR (aerobic exercise[tiab])) OR ("resistance training"[Mesh]) OR ("walking"[Mesh]))) AND ((randomized controlled trial[pt] OR controlled clinical trial[pt] OR randomized[tiab] OR placebo[tiab] OR randomly[tiab] OR trial[tiab] OR groups[tiab]) NOT (animals[mh] NOT humans[mh]))

**Web of science:**

**Table 1** Search strategy for the Web of Science

| Number | Search items |
|---|---|
| 1 | TS=(breast neoplasms OR breast cancer OR breast tumor OR mammary cancer OR breast carcinoma) |
| 2 | TS=(sleep initiation and maintenance disorders OR insomnia) |
| 3 | TS=(complementary therapies OR complementary medicine OR alternative medicine OR alternative therapies OR integrative medicine |
| 4 | TS=(acupuncture OR electroacupuncture OR auricular acupuncture OR tuina OR massage) |
| 5 | TS=(cognitive behavioral therapy OR mindfulness OR meditation) |
| 6 | TS=(mind-body therapies) |
| 7 | TS=(yoga) |

| | |
|---|---|
| **8** | TS=(tai ji OR tai chi) |
| **9** | TS=(qigong) |
| **10** | TS=(exercise therapy OR physical exercise OR aerobic exercise) |
| **11** | TS=(resistance training) |
| **12** | TS=(walking) |
| **13** | TS=(randomized controlled trial OR controlled clinical trial OR randomized OR placebo OR clinical trials as topic OR randomly OR trial) |
| **14** | TS=(animals) |
| **15** | TS=(humans AND animals) |
| **16** | ((#13) NOT #14) NOT #15 |
| **17** | #3 OR #4 OR #5 OR #6 OR #7 OR #8 OR #9 OR #10 OR #11 OR #12 |
| **18** | #1 AND #2 AND #16 AND #17 |

**Medline:**

1. breast neoplasms[Mesh] or (breast cancer or breast tumor or mammary cancer or breast carcinoma).ti,ab.
2. (sleep initiation and maintenance disorders).mp. or (insomnia).ti,ab.
3. complementary therapies[Mesh] or (complementary medicine or alternative medicine or alternative therapies or integrative medicine).ti,ab.
4. acupuncture[Mesh] or (electroacupuncture or auricular acupuncture).ti,ab.
5. massage[Mesh] or (tuina).ti,ab.
6. cognitive behavioral therapy[Mesh]
7. mindfulness[Mesh]
8. meditation[Mesh]
9. mind-body therapies[Mesh]
10. yoga[Mesh]
11. tai ji[Mesh] or (tai chi).ti,ab.
12. qigong[Mesh]
13. exercise[Mesh] or (physical exercise or aerobic exercise).ti,ab.
14. resistance training[Mesh]

15. walking[Mesh]

16. (randomized controlled trial or controlled clinical trial).pt. or random$.ab. or trial.ab.

17. 3 or 4 or 5 or 6 or 7 or 8 or 9 or 10 or 11 or 12 or 13 or 14 or 15

18. 1 and 2 and 16 and 17

**Embase：**

1. 'breast neoplasms'/exp OR 'breast neoplasms' OR 'breast cancer':ab,ti OR 'breast tumor':ab,ti OR 'mammary cancer':ab,ti OR 'breast carcinoma':ab,ti

2. 'sleep initiation and maintenance disorder'/exp OR 'sleep initiation and maintenance disorder' OR 'insomnia':ab,ti

3. 'complementary therapies'/exp OR 'complementary therapies' OR 'complementary medicine':ab,ti OR 'alternative medicine':ab,ti OR 'alternative therapies':ab,ti OR 'integrative medicine':ab,ti

4. 'acupuncture'/exp OR 'acupuncture' OR 'electroacupuncture':ab,ti OR 'auricular acupuncture':ab,ti

5. 'massage'/exp OR 'massage' OR 'tuina':ab,ti

6. 'cognitive behavioral therapy'/exp OR 'cognitive behavioral therapy'

7. 'mindfulness'/exp OR 'mindfulness'

8. 'meditation'/exp OR 'meditation'

9. 'mind-body therapies'/exp OR 'mind-body therapies'

10. 'yoga'/exp OR 'yoga'

11. 'tai ji'/exp OR 'tai ji' OR 'tai chi':ab,ti

12. 'qigong'/exp OR 'qigong'

13. 'exercise'/exp OR 'exercise' OR 'physical exercise':ab,ti OR 'aerobic exercise':ab,ti

14. 'resistance training'/exp OR 'resistance training'

15. 'walking'/exp OR 'walking'

16. 'crossover procedure':de OR 'double-blind procedure':de OR 'randomized controlled trial':de OR 'single-blind procedure':de OR (random* OR factorial* OR crossover* OR cross NEXT/1 over* OR placebo* OR doubl* NEAR/1 blind* OR singl* NEAR/1 blind* OR assign* OR allocat* OR volunteer*):de,ab,ti

17. 3 or 4 or 5 or 6 or 7 or 8 or 9 or 10 or 11 or 12 or 13 or 14 or 15

18. 1 and 2 and 16 and 17

**Cochrane：**

#1 ((breast neoplasms) OR (breast cancer) OR (breast tumor) OR (mammary cancer) OR (breast carcinoma)):ab,ti,kw

#2 (sleep initiation and maintenance disorders) OR (insomnia):ab,ti,kw

#3 (((complementary therapies) OR (complementary medicine) OR (alternative medicine) OR (alternative therapies) OR (integrative medicine)) OR ((acupuncture) OR (electroacupuncture) OR (auricular acupuncture) OR (tuina) OR (massage)) OR ((cognitive behavioral therapy) OR (mindfulness) OR (meditation)) OR (mind-body therapies) OR (yoga) OR ((tai ji) OR (tai chi)) OR (qigong) OR ((exercise therapy) OR (physical exercise) OR (aerobic exercise)) OR (resistance training) OR (walking)):ab,ti,kw

#1 AND #2 AND #3

**CBM：**

("失眠"[摘要] OR "不寐"[摘要] OR "睡眠障碍"[摘要] OR "睡眠"[摘要]) AND ("乳腺癌"[摘要] OR "乳腺肿瘤"[摘要]) AND ("补充医学"[摘要] OR "替代医学"[摘要] OR "结合医学"[摘要] OR "针刺"[摘要] OR "针灸"[摘要] OR "电针"[摘要] OR "耳针"[摘要] OR "推拿"[摘要] OR "按摩"[摘要] OR "正念"[摘要] OR "冥想"[摘要] OR "认知行为"[摘要] OR "瑜伽"[摘要] OR "太极"[摘要] OR "气功"[摘要] OR "有氧"[摘要] OR "运动"[摘要] OR "体育锻炼"[摘要] OR "身心"[摘要] OR "抗阻"[摘要])

**CNKI：**

(TKA = '失眠' OR TKA = '不寐' OR TKA = '睡眠' OR TKA = '睡眠障碍') AND (TKA = '乳腺癌' OR TKA = '乳腺肿瘤') AND (TKA = '补充医学' OR TKA = '替代医学' OR TKA = '结合医学' OR TKA = '针刺' OR TKA = '针灸' OR TKA = '电针' OR TKA = '耳针' OR TKA = '推拿' OR TKA = '按摩' OR TKA = '正念' OR TKA = '冥想' OR TKA = '认知行为' OR TKA = '瑜伽' OR TKA = '太极' OR TKA = '气功'

OR TKA = '有氧' OR TKA = '运动' OR TKA = '体育锻炼' OR TKA = '身心' OR TKA = '抗阻')

**VIP：**

M=(失眠 OR 不寐 OR 睡眠 OR 睡眠障碍) AND M=(乳腺癌 OR 乳腺肿瘤) AND M=(补充医学 OR 替代医学 OR 结合医学 OR 针刺 OR 针灸 OR 电针 OR 耳针 OR 推拿 OR 按摩 OR 正念 OR 冥想 OR 认知行为 OR 瑜伽 OR 太极 OR 气功 OR 有氧 OR 运动 OR 体育锻炼 OR 身心 OR 抗阻)

**WANFANG：**

题名或关键词:(("失眠" or "不寐" or "睡眠" or "睡眠障碍") and ("乳腺癌" or "乳腺肿瘤") and ("补充医学" or "替代医学" or "结合医学" or "针刺" or "针灸" or "电针" or "耳针" or "推拿" or "按摩" or "正念" or "冥想" or "认知行为" or "瑜伽" or "太极 " or "气功" or "有氧" or "运动" or "体育锻炼" or "身心" or "抗阻"))
