# Supplementary material for: Non-pharmaceutical interventions in complementary and alternative medicine for insomnia in breast cancer survivors: a protocol for a systematic review and network meta-analysis
Source: BMJ Open. 2023 May 4;13(5):e071784. doi: 10.1136/bmjopen-2023-071784 (PMC10163449; doi:10.1136/bmjopen-2023-071784)
Supplement: Author's manuscript [file bmjopen-2023-071784.draft_revisions.pdf]

BMJ Open is committed to open peer review. As part of this commitment we make the peer review history of every article we publish publicly available.

When an article is published we post the peer reviewers' comments and the authors' responses online. We also post the versions of the paper that were used during peer review. These are the versions that the peer review comments apply to.

The versions of the paper that follow are the versions that were submitted during the peer review process. They are not the versions of record or the final published versions. They should not be cited or distributed as the published version of this manuscript.

BMJ Open is an open access journal and the full, final, typeset and author-corrected version of record of the manuscript is available on our site with no access controls, subscription charges or pay-per-view fees (http://bmjopen.bmj.com).

If you have any questions on BMJ Open's open peer review process please email info.bmjopen@bmj.com

BMJ Open

Complementary and alternative medicine for insomnia in breast cancer survivors: a protocol for a systematic review and network meta-analysis

Journal:	*BMJ Open*
Manuscript ID	bmjopen-2023-071784
Article Type:	Protocol
Date Submitted by the Author:	10-Jan-2023
Complete List of Authors:	Jin, Qi; Shanghai Municipal Hospital of Traditional Chinese Medicine<br>LIU, LUMIN; Long Hua Hospital<br>Chen, Yue-lai; Long Hua Hospital<br>Yin, Ping; Long Hua Hospital
Keywords:	COMPLEMENTARY MEDICINE, Breast tumours < ONCOLOGY, Quality in health care < HEALTH SERVICES ADMINISTRATION & MANAGEMENT

SCHOLARONE™
Manuscripts

Title Page

Title: Complementary and alternative medicine for insomnia in breast cancer survivors: a protocol for a systematic review and network meta-analysis

Author information:

The full names of authors: Qi Jin, Lumin Liu, Yuelai Chen, Ping Yin

The affiliation(s) and address(es) of the author(s):

Qi Jin, Shanghai Municipal Hospital of Traditional Chinese Medicine, Shanghai University of Traditional Chinese Medicine, Shanghai 200071, China.

Email: jessyqi0606@163.com.

Lumin Liu, LongHua Hospital Shanghai University of Traditional Chinese Medicine, Shanghai 200032, China.

Email: beefllm@163.com.

Yuelai Chen, LongHua Hospital Shanghai University of Traditional Chinese Medicine, Shanghai 200032, China.

Email: chenyuelai@163.com.

Ping Yin, LongHua Hospital Shanghai University of Traditional Chinese Medicine, Shanghai 200032, China.

Email: bingxue616@163.com.

Co-first authors:

Qi Jin and Lumin Liu contributed equally to this article.

Corresponding author:

Dr. *Ping Yin*, LongHua Hospital Shanghai University of Traditional Chinese Medicine, Shanghai 200032, China. Email: bingxue616@163.com.

Professor *Yuelai Chen*, LongHua Hospital Shanghai University of Traditional Chinese Medicine, Shanghai 200032, China. Email: chenyuelai@163.com.

For peer review only - http://bmjopen.bmj.com/site/about/guidelines.xhtml

Complementary and alternative medicine for insomnia in breast cancer survivors: a protocol for a systematic review and network meta-analysis

Qi Jin[2], Lumin Liu[1], Yuelai Chen[1], Ping Yin[1]

Author affiliations

[1.] LongHua Hospital Shanghai University of Traditional Chinese Medicine, Shanghai, China.

[2.] Shanghai Municipal Hospital of Traditional Chinese Medicine, Shanghai University of Traditional Chinese Medicine, Shanghai, China.

ABSTRACT

Introduction Insomnia has a high incidence in breast cancer patients, which not only affects the quality of life of patients, but also affects the efficiency of later treatment and rehabilitation. Although the sedative and hypnotic drugs commonly used in clinical practice have a rapid onset of action, they are also accompanied by different degrees of sequelae, withdrawal effects and dependence and addiction. Complementary and alternative medicine (CAM), as a non-pharmacological treatment, including acupuncture, behavioral intervention, physical exercise, etc., has been reported by a large number of studies and recommended for the treatment of cancer-related insomnia. Its clinical results are increasingly recognized and accepted by patients. However, the effectiveness and safety of these CAM are uneven, and there is no standard clinical application method. Therefore, in order to objectively evaluate the effects of different CAM on insomnia, we will conduct a network meta-analysis to explore the effects of different CAM interventions on improving sleep quality in breast cancer patients.

Methods and analysis We will search for all publications published in English and Chinese from 2000 until 10 December 2022. The databases include PubMed, Medline, Embase, Web of Science and Cochrane Central Register of Controlled Trials, and the Chinese literature databases include CBM, CNKI, VIP, WANFANG. The insomnia

For peer review only - http://bmjopen.bmj.com/site/about/guidelines.xhtml

severity index (ISI) and the Pittsburgh sleep quality index (PSQI) will be considered as the primary outcomes in the study. STATA V.15.0 software will be used to conduct pairwise meta-analysis and NMA. Finally, We will use the recommended risk assessment tool ROB2 for risk and bias assessment, and use the GRADE grading evaluation method to evaluate the quality of evidence.

Ethics and dissemination Ethical approval will not be required because the study will not include the original information of participants. The results will be published in a peer-reviewed journal or disseminated in relevant conferences.

PROSPERO registration number CRD42022382602.

INTRODUCTION

Insomnia, as one of the most prevalent complaints among cancer survivors, mainly manifests as difficulty in initiating and/or maintaining sleep, early morning awakening, which are associated with impaired performance and daytime well being.[1] Insomnia is common in most cancers but appears particularly in breast cancer. According to the epidemiological investigation, 36%-59% patients experienced insomnia during the cancer trajectories. Among them, rates were greater in patients with breast cancer from 42% to 69%.[2] In a 5-year follow-up investigation, 38% disease-free breast cancer survivors reported that they still had sleep problems.[3] Insomnia in breast cancer survivors can be debilitating, resulting in adverse impact on patients' life quality, and exacerbation on other symptoms, including pain, fatigue, hot flash and mental disorders.[4] Moreover, insomnia was associated with poor survival rate for patients with breast cancer.[5]

Currently the interventions in treating breast cancer related insomnia include pharmaceutical therapy and non-pharmaceutical ones. Due to the drug resistance, as well as the psychological and physical dependence, complementary and alternative medicine (CAM) has begun to receive more and more attention. Cognitive behavioral therapy for insomnia (CBT-I) has been demonstrated as an effective treatment and wildly recommended for breast cancer survivors.[6–7] A growing concern about the efficacy of acupuncture therapy showed that it can improve sleep quality with mental

For peer review only - http://bmjopen.bmj.com/site/about/guidelines.xhtml

and emotional distress symptoms.[8–9] Mind-body therapies including yoga, taichi or qigong also seem to be available for breast cancer related insomnia.[10–11] Besides, several studies reported that physical exercise can relieve the insomnia disorder of breast cancer patients.[12–13] In conclusion, previous pairwise meta-analysis focused on only one CAM intervention mentioned above has demonstrated the efficacy in breast cancer related to insomnia.

However, apparent uncertainty about which non-pharmaceutical intervention is the most effective option makes it difficult for clinicians to manage this troublesome situation. In a randomized controlled trail, CBT-I showed superiority in shortening sleep onset latency, reducing wake, improving sleep efficiency while acupuncture was better at increasing total sleep time. Besides, the differences between acupuncture and CBT-I for women, non-whites, lower education, and those with pain were insignificant under subgroup analysis.[14] Thus, a comparison of these non-pharmaceutical interventions and the relative effectiveness between them represents an impending need for patients with breast cancer.

Network meta-analysis (NMA), which is also named multiple treatments comparison meta-analysis, helps to combine the direct and indirect evidence across a network of treatments. Furthermore, NMA is able to provide the ranking of treatment options based on their effectiveness.[15] However, to the best of our knowledge, so far NMA hasn't been used to perform the above functions in treating breast cancer related insomnia. Therefore, by the mean of systematic review (SR) and NMA, this study aims to compare the efficacy and evaluate the safety of different CAM interventions for breast cancer survivors with insomnia.

METHODS

Study registration

This protocol was registered on PROSPERO (CRD42022382602) and was reported following the Preferred Reporting Items for Systematic Review and Meta-Analysis Protocols (PRISMA-P) statement guidelines (online supplemental file 1 for PRISMA-P checklist). The findings of this study will be presented following the

For peer review only - http://bmjopen.bmj.com/site/about/guidelines.xhtml

checklist of items to include when reporting a systematic review involving a network meta-analysis (PRISMA-NMA)

Inclusion criteria

Types of studies

We will incorporate RCTs that include multiple and single branches designs, full-text journal publications, unpublished clinical trials with online results in Chinese and English, and RCTs conducted in any healthcare setting.

Participants

Studies enrolled participants (18 years and older) who were diagnosed with breast cancer (stage 0-IV) and insomnia by any standardized diagnostic tool. Patients who are undergoing or have completed chemotherapy or surgery. There will be no restrictions on race, nationality, and education.

Types of interventions

CAM interventions, as the main treatment, will be included. CAM interventions will be limited to several aspects of the following four treatments. (1)CBT-I which has only one component of sleep restriction, stimulus control, relaxation training and meditation (including at least 2 components of cognitive therapy, stimulus control, sleep hygiene (including sleep education), sleep restriction, relaxation training and other variants of CBT-I, and mono-behavioral therapy);(2) Acupuncture (including acupuncture, electroacupuncture, auricular acupuncture); (3) Mind-body exercise (including yoga, tai chi and Qigong); (4)Physical exercise (including aerobic exercise, resistance exercise, walking, and interventions increasing physical activities). Studies used single or multiple complementary alternative medical intervention(s) will be considered.

Types of comparator(s)/control

The comparison group will be included usual care, a waiting control, placebo, sham acupuncture and sleep education.

Primary outcomes

The insomnia severity index (ISI) and the Pittsburgh sleep quality index (PSQI)

For peer review only - http://bmjopen.bmj.com/site/about/guidelines.xhtml

will be considered as the primary outcomes in the study.

The ISI consists of 7 items to assess the nature and symptoms of the subject's sleep disorders. Questions address subjects' subjective ratings of sleep quality, including the severity of symptoms, subjects' satisfaction with their sleep patterns, the impact of the degree of insomnia on daily functioning, subjects' awareness of the impact of insomnia on themselves, and their levl of frustration due to their sleep disorders.[16]

PSQI instrument produces a global sleep quality score and seven specific component scores: quality, latency, duration, disturbance, habitual sleep efficiency, use of sleeping medications, and daytime dysfunction. Global scores, ranging from 0 to 21 with higher scores, indicates poor sleep quality and high sleep disturbance.[17]

Secondary outcomes

Sleep efficiency, sleep latency (SOL), wake-up time after sleep onset (WASO), and total sleep time (TST) derived from polysomnography data or sleep diaries will be regarded as secondary outcome measures. In addition, quantitative statistics of adverse events as another secondary indicator.[18]

Exclusion criteria

1. Studies that are duplicates.
2. Patients with other diseases that affect insomnia.
3. The design type is non- RCT, including conference paper, summary, abstracts, editorials, clinical observations, case studies, cohort studies, non-randomized trials, case-control studies, any non-experimental investigation (including cross-sectional and retrospective surveys).
4. We generally exclude studies involving various types of cancer unless they have subgroup data for breast cancer.
5. The full text of the study is not available.

Any study that meets one or more of the above criteria will not be considered.

Search methods for identification of studies

We will search for all publications published in English and Chinese from 2000 until 10 December 2022. The English literature databases include PubMed, Medline,

For peer review only - http://bmjopen.bmj.com/site/about/guidelines.xhtml

Embase, Web of Science and Cochrane Central Register of Controlled Trials, and the Chinese literature databases include CBM, CNKI, VIP, WANFANG. The search keywords were focused on ' breast cancer ', ' insomnia ', ' acupuncture ', ' electroacupuncture ', ' Auricular acupuncture ', ' cognitive behavioral therapy ', ' Mind-body therapies ', ' yoga ', ' tai chi ', ' qigong ', ' physical exercise ', ' resistance training ',' Walking ',' randomized controlled trial '. The final search formula was obtained by searching for the subject words, subordinate words and free words. The reference list of all selected articles will be independently screened to identify additional studies missed in the initial search. Table 1 shows specific search strategies for Web of science.

Table 1 Search strategy for the Web of Science

Number	Search items
#1	TS=(Breast neoplasms OR Breast cancer OR Breast Tumor OR Mammary cancer OR Breast Carcinoma)
#2	TS=(sleep initiation and maintenance disorders OR insomnia)
#3	TS=(Acupuncture OR Electroacupuncture OR Auricular acupuncture)
#4	TS=(cognitive behavioral therapy)
#5	TS=(Mind-body therapies)
#6	TS=(Yoga)
#7	TS=(Tai ji OR Tai Chi)
#8	TS=(qigong)
#9	TS=(exercise therapy OR Physical exercise OR Aerobic exercise)
#10	TS=(resistance training)
#11	TS=(Walking)
#12	TS=(randomized controlled trial OR controlled clinical trial OR randomized OR placebo OR clinical trials as topic OR randomly OR trial)
#13	TS=(animals)
#14	TS=(humans AND animals)
#15	((#12) NOT #13) NOT #14

For peer review only - http://bmjopen.bmj.com/site/about/guidelines.xhtml

**#16**	#3 OR #4 OR #5 OR #6 OR #7 OR #8 OR #9 OR #10 OR #11
**#17**	#1 AND #2 AND #16 AND #15

Data collection and analysis

Selection of studies

Two researchers (QJ and LML) will screen the literature independently and extract the data and cross-check them. If there are differences, these will be resolved through discussion or negotiation with third parties. In the literature screening, the title of the article will be first read. After excluding obviously unrelated literatures, the abstract and full text will be further read to determine whether it will be included. If necessary, the authors of the original studies will be contacted by email or telephone to obtain information that has not been identified but is important to this study. EndnoteX9 will be used to manage retrieved studies and remove duplicates. The selection procedure is presented in a PRISMA flow chart (Figure 1).

Data extraction and management

Two researchers (QJ and LML) will independently extract data based on a predesigned form. The extracted data will be as follows:(1) Basic information included in the study: first author and year of publication; (2) Research methods: research type, intervention measures, control measures, sample size; (3) Concerned Outcome indicators and outcome measurement data. (4) Follow-up. The corresponding authors will be contacted for missing information. The two researchers will crosscheck the data after completion of data extraction. The disagreements will be solved by the team discussion or consultation with the third researcher (YLC).

Quality assessment

Two independent reviewers (QJ and PY) will complete the risk-of-bias assessment and evaluation of evidence certainty.

We will use Version 2 of the Cochrane tool for assessing the risk of bias in randomized trials (RoB2) revised by the Cochrane Methodology Group in 2019.[19] The quality of the study will be evaluated in the following five areas: (1) bias in the

For peer review only - http://bmjopen.bmj.com/site/about/guidelines.xhtml

randomization process, (2) deviation from established interventions, (3) bias in missing outcome data, (4) bias in outcome measurement, and (5) selective reporting of bias in results.

The Grading of Recommendations Assessment, Development and Evaluation System (GRADE) system will be used to grade the quality of the evidence for main outcomes.[20] Evidence quality will be rated 'high', 'moderate', 'low' or 'very low' according to the GRADE rating standards. The evidence quality of a specific study will be assessed according to the risk of bias, inconsistency, indirectness, imprecision and publication bias.

Pairwise meta-analysis

The pairwise meta-analysis will be performed using STATA 15.0. Conventional pairwise meta-analyses will be performed with random-effects model. Pooled odds ratio (OR) with 95% credible intervals (CIs) will be used for the dichotomous variables. Standardized mean differences (SMDs) or weighted mean differences (WMDs) with 95% CIs will be used for the continuous variables. The heterogeneity between trials will be estimated using I^2 statistics and P value. The values of 25%, 50%, and 75% for the I^2 as indicative of low, moderate, and high statistical heterogeneity, respectively. We will explore sources of heterogeneity by subgroup analysis and meta-regression.[21]

Network meta-analysis

If enough data is available, we will conduct a network meta-analysis (NMA) to compare the efficacy of different interventions and controls. The NMA will be conducted on both direct evidence and indirect evidence in a Bayesian framework using STATA V.15.0 software. The publication bias will be evaluated with the Egger's test and funnel plots if the number of studies exceeds. A 2-tailed P value < 0.05 is considered as statistically significant.[22] In addition, a node-splitting model will also be used to assess local inconsistency at the network level, and a consistency or inconsistency model will be selected based on the results. If P<0.05, there was a statistically significant difference between the indirect and direct multiple treatment comparisons. To evaluate the convergence of the results, we will analyze the potential scale reduction factor (PSRF), where a PSRF value close to 1 indicates successful

For peer review only - http://bmjopen.bmj.com/site/about/guidelines.xhtml

convergence.[23]

Subgroup analysis, meta regression analysis and sensitivity analysis

Subgroup analysis and meta-regression analysis will be used to explore possible sources of heterogeneity and inconsistency. Subgroup analysis will be performed according to the different types of non-drug interventions. We will conduct a meta-regression analysis of participants who completed chemotherapy or radiotherapy for more than 6 months and those who did not complete chemotherapy or radiotherapy for less than 6 months as covariates. In addition, the sensitivity analysis is carried out by the method of exclusion to verify the robustness of the results.

Publication bias assessment

If more than 10 studies are included, a comparison-adjusted funnel plot will be generated to detect reporting bias.

Patients and public involvement

This is a SR and NMA based on existing studies, so no patients or members of the public will directly be involved.

DISCUSSION

Breast cancer is the most common malignancy among women. According to the 2020 Global burden of cancer data released by the International Agency for Research on Cancer (IARC) of the World Health Organization, there are as many as 2.26 million new cases of breast cancer worldwide.[24] About 40% of breast cancer patients have insomnia.[25] Even though sleeping pills are often prescribed for insomnia, they can lead to drug dependence and other side effects.[26] Moreover, the use of sleeping pills in cancer patients may interfere with the original cancer chemotherapy regimen and even cause the disease to worsen.[27] CAM refers to the approaches and methods of diagnosis and treatment outside the mainstream of traditional western medicine.[28] In the advancement of society, the number of people receiving and benefiting from CAM is increasing, and numerous clinical studies have been conducted to prove the effectiveness and safety of CAM.[29–30]

In previous studies, there is only one SR report on physical exercise and mind-

For peer review only - http://bmjopen.bmj.com/site/about/guidelines.xhtml

body exercise in the treatment of insomnia in breast cancer, and there is no specific SR study on insomnia in breast cancer patients for other CAM therapies.[10] According to our knowledge, this may be the first SR and NMA study to compare the efficacy and safety of complementary replacement therapy in breast cancer insomnia survivors according to ISI, PSQI, and sleep diary related measures. The results of the study will provide a ranking of different complementary alternative therapy interventions to assist patients, physicians, and policy makers in their decision making. Further, GRADE will be used for evaluating the evidence's certainty. However, the study also has some limitations. First, complementary and alternative therapy interventions for insomnia in breast cancer are an extensive area of research, but our focus on acupuncture, CBT-I, and exercise (both mind-body and physical exercise) may lead to limitations in clinical practice. Second, the study will not examine the effects of different regimens of the same complementary and alternative therapy intervention.

Ethics and dissemination

The study will not require ethical approval because it comprises analysis based on existing studies. The results are expected to be published in a peer-reviewed journal or disseminated at relevant conferences. The objective of our study will evaluate the impact of complementary alternative therapies (including acupuncture, CBT-I, physical exercise, mind-body exercise etc.) on sleep-related outcomes in breast cancer survivors with insomnia by conducting a systematic review and network meta-analysis of existing randomized controlled trial data for the purpose of providing reliable evidence-based medical evidence for complementary and alternative therapies for insomnia in breast cancer.

Contributors

QJ and LML contributed equally to this article as co-first authors. YLC and PY contributed to the design of the study. The manuscript was drafted by QJ and LML. QJ and LML participated in the design of the search strategy and data extraction dataset. YLC and PY will monitor each procedure of the review and be responsible for quality control. All the authors participated in the reading, discussion and revision of the manuscript, and all approved of the protocol for publication.

For peer review only - http://bmjopen.bmj.com/site/about/guidelines.xhtml

Funding

This work was supported by the Clinical Incubation Program of the National Medical Center of LongHua Hospital to Shanghai University of Traditional Chinese Medicine, with grant number (GY202201).

Competing interests

None declared.

Patient and public involvement

Patients and/or the public were not involved in the design, conduct, reporting, or dissemination plans of this research.

Patient consent for publication

Not applicable.

Provenance and peer review

Not commissioned; externally peer reviewed.

References

[1] Schieber K, Niecke A, Geiser F, *et al*. The course of cancer-related insomnia: don't expect it to disappear after cancer treatment. *Sleep Med* 2019;58:107-113.

[2] Savard J, Ivers H, Villa J, *et al*. Natural course of insomnia comorbid with cancer: an 18-month longitudinal study. *J Clin Oncol* 2011;29(26):3580-3586.

[3] Schmidt ME, Wiskemann J, Steindorf K. Quality of life, problems, and needs of disease-free breast cancer survivors 5 years after diagnosis. *Qual Life Res* 2018;27(8):2077-2086.

[4] Collins KP, Geller DA, Antoni M, *et al*. Sleep duration is associated with survival in advanced cancer patients. *Sleep Med* 2017;32:208-212.

[5] Costa AR, Fontes F, Pereira S, *et al*. Impact of breast cancer treatments on sleep disturbances - A systematic review. *Breast* 2014;23(6):697-709.

[6] Ma Y, Hall DL, Ngo LH, *et al*. Efficacy of cognitive behavioral therapy for insomnia in breast cancer: A meta-analysis. *Sleep Med Rev* 2021;55:101376.

[7] Bean HR, Diggens J, Ftanou M, *et al*. Light enhanced cognitive behavioral therapy for insomnia and fatigue during chemotherapy for breast cancer: a randomized controlled trial. *Sleep* 2022;45(3).

[8] Zhang JL, Zhang ZN, Huang ST, *et al*. Acupuncture for cancer-related insomnia: A systematic review and meta-analysis. *Phytomedicine* 2022;102:154160.

[9] Garland SN, Xie SX, Li Q, *et al*. Comparative effectiveness of electro-acupuncture versus gabapentin for sleep disturbances in breast cancer survivors with hot flashes: a randomized trial. *Menopause* 2017;24(5):517-523.

[10] Kreutz C, Schmidt ME, Steindorf K. Effects of physical and mind-body exercise on sleep problems

For peer review only - http://bmjopen.bmj.com/site/about/guidelines.xhtml

during and after breast cancer treatment: a systematic review and meta-analysis. *Breast Cancer Res Treat* 2019;176(1):1-15.

[11] Chaoul A, Milbury K, Spelman A, *et al*. Randomized trial of Tibetan yoga in patients with breast cancer undergoing chemotherapy. *Cancer* 2018;124(1):36-45.

[12] Mercier J, Savard J, Bernard P. Exercise interventions to improve sleep in cancer patients: A systematic review and meta-analysis. *Sleep Med Rev* 2017;36:43-56.

[13] Steindorf K, Wiskemann J, Ulrich CM, *et al*. Effects of exercise on sleep problems in breast cancer patients receiving radiotherapy: a randomized clinical trial. *Breast Cancer Res Treat* 2017; 162 (3):489-499.

[14] Garland SN, Xie SX, DuHamel K, *et al*. Acupuncture Versus Cognitive Behavioral Therapy for Insomnia in Cancer Survivors: A Randomized Clinical Trial. *J Natl Cancer Inst* 2019;111(12):1323-1331.

[15] Bafeta A, Trinquart L, Seror R, *et al*. Reporting of results from network meta-analyses: methodological systematic review. *BMJ* 2014;348:g1741.

[16] Morin CM, Belleville G, Bélanger L, *et al*. The Insomnia Severity Index: psychometric indicators to detect insomnia cases and evaluate treatment response. *Sleep* 2011;34(5):601-608.

[17] Buysse DJ, Reynolds CR, Monk TH, *et al*. The Pittsburgh Sleep Quality Index: a new instrument for psychiatric practice and research. *Psychiatry Res* 1989;28(2):193-213.

[18] Fekedulegn D, Andrew ME, Shi M, *et al*. Actigraphy-Based Assessment of Sleep Parameters. *Ann Work Expo Health* 2020;64(4):350-367.

[19] Sterne J, Savović J, Page MJ, *et al*. RoB 2: a revised tool for assessing risk of bias in randomised trials. *BMJ* 2019;366:l4898.

[20] Guyatt GH, Oxman AD, Vist GE, *et al*. GRADE: an emerging consensus on rating quality of evidence and strength of recommendations. *BMJ* 2008;336(7650):924-926.

[21] Higgins JP, Thompson SG, Deeks JJ, *et al*. Measuring inconsistency in meta-analyses. *BMJ* 2003;327(7414):557-560.

[22] Salanti G, Ades AE, Ioannidis JP. Graphical methods and numerical summaries for presenting results from multiple-treatment meta-analysis: an overview and tutorial. *J Clin Epidemiol* 2011;64 (2):163-171.

[23] A GB. General Methods for Monitoring Convergence of Iterative Simulations. *Journal of Computational & Graphical Statistics* 1998;4(7):434-455.

[24] Sung H, Ferlay J, Siegel RL, *et al*. Global Cancer Statistics 2020: GLOBOCAN Estimates of Incidence and Mortality Worldwide for 36 Cancers in 185 Countries. *CA Cancer J Clin* 2021;71(3):209 -249.

[25] Leysen L, Lahousse A, Nijs J, *et al*. Prevalence and risk factors of sleep disturbances in breast cancersurvivors: systematic review and meta-analyses. *Support Care Cancer* 2019;27(12):4401-4433.

[26] Williams F, Jeanetta SC. Lived experiences of breast cancer survivors after diagnosis, treatment and beyond: qualitative study. *Health Expect* 2016;19(3):631-642.

[27] Sivertsen B, Salo P, Pentti J, *et al*. Use of sleep medications and risk of cancer: a matched case-control study. *Sleep Med* 2015;16(12):1552-1555.

[28] Alsharif F. Discovering the Use of Complementary and Alternative Medicine in Oncology Patients: A Systematic Literature Review. *Evid Based Complement Alternat Med* 2021;2021:6619243.

[29] Ng JY, Sharma AE. Guidelines for Cancer-Related Pain: A Systematic Review of Complementary and Alternative Medicine Recommendations. *Pain Pract* 2021;21(4):454-467.

For peer review only - http://bmjopen.bmj.com/site/about/guidelines.xhtml

[30] Mora DC, Kristoffersen AE, Overvåg G, *et al*. Safety of Complementary and Alternative Medicine (CAM) treatment among children and young adults who suffer from adverse effects of conventional cancer treatment: A systematic review. *Integr Cancer Ther* 2022;21:1553420699.

For peer review only

For peer review only - http://bmjopen.bmj.com/site/about/guidelines.xhtml

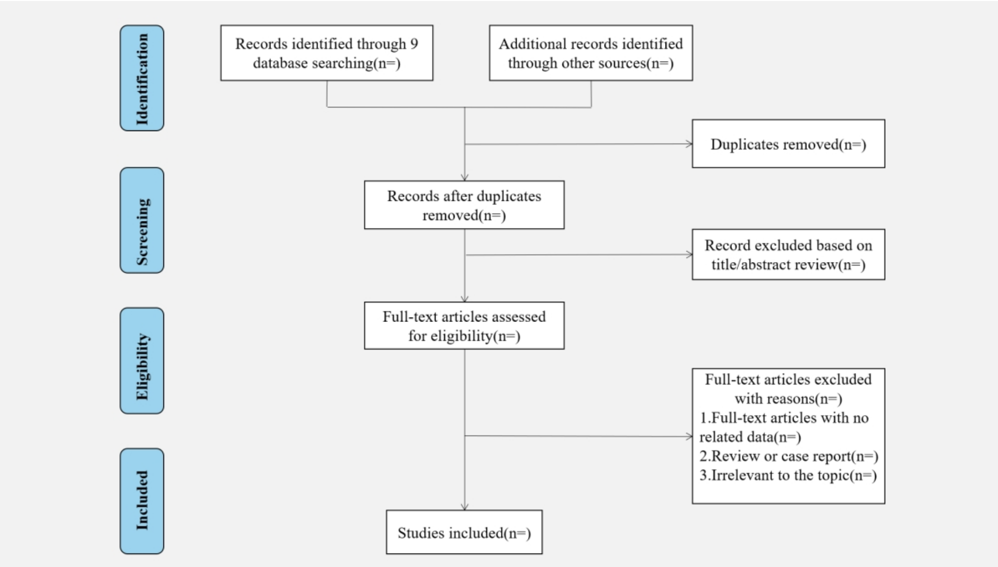

Figure 1 PRISMA flow diagram of the study selection process

276x167mm (192 x 192 DPI)

PRISMA-P (Preferred Reporting Items for Systematic review and Meta-Analysis Protocols) 2015 checklist: recommended items to address in a systematic review protocol*

Section and topic	Item No	Checklist item	Location in the article
**ADMINISTRATIVE INFORMATION**			
Title:			
Identification	1a	Identify the report as a protocol of a systematic review	Page 1, Lines 2-3
Update	1b	If the protocol is for an update of a previous systematic review, identify as such	NA
Registration	2	If registered, provide the name of the registry (such as PROSPERO) and registration number	Page 4 , Line 28
Authors:			
Contact	3a	Provide name, institutional affiliation, e-mail address of all protocol authors; provide physical mailing address of corresponding author	Page 1 , Lines 6-29
Contributions	3b	Describe contributions of protocol authors and identify the guarantor of the review	Page 11 , Lines 29-30<br>Page 12 , Lines 1-5
Amendments	4	If the protocol represents an amendment of a previously completed or published protocol, identify as such and list changes; otherwise, state plan for documenting important protocol amendments	NA
Support:			
Sources	5a	Indicate sources of financial or other support for the review	Page 12 , Lines 6-9
Sponsor	5b	Provide name for the review funder and/or sponsor	Page 12 , Lines 6-9
Role of sponsor or funder	5c	Describe roles of funder(s), sponsor(s), and/or institution(s), if any, in developing the protocol	NA
**INTRODUCTION**			
Rationale	6	Describe the rationale for the review in the context of what is already known	Page 3 , Lines 12-30<br>Page 4 , Lines 1-21
Objectives	7	Provide an explicit statement of the question(s) the review will address with reference to participants, interventions, comparators, and outcomes (PICO)	Page 4 , Lines 22-24
**METHODS**			
Eligibility criteria	8	Specify the study characteristics (such as PICO, study design, setting, time frame) and report characteristics (such as years considered, language, publication status) to be used as criteria for eligibility for the review	Page 5 , Lines 5-29<br>Page 6 , Lines 1-28
Information sources	9	Describe all intended information sources (such as electronic databases, contact with study authors, trial registers or other grey literature sources) with planned dates of coverage	Page 7 , Lines 1-12

For peer review only

Search strategy	10	Present draft of search strategy to be used for at least one electronic database, including planned limits, such that it could be repeated	Page 7 , Table1
Study records:			
Data management	11a	Describe the mechanism(s) that will be used to manage records and data throughout the review	Page 8 , Line 10
Selection process	11b	State the process that will be used for selecting studies (such as two independent reviewers) through each phase of the review (that is, screening, eligibility and inclusion in meta-analysis)	Page 8 , Lines 3-9
Data collection process	11c	Describe planned method of extracting data from reports (such as piloting forms, done independently, in duplicate), any processes for obtaining and confirming data from investigators	Page 8 , Lines 18-20
Data items	12	List and define all variables for which data will be sought (such as PICO items, funding sources), any pre-planned data assumptions and simplifications	Page 8 , Lines 14-17
Outcomes and prioritization	13	List and define all outcomes for which data will be sought, including prioritization of main and additional outcomes, with rationale	Page 6 , Lines 1-17
Risk of bias in individual studies	14	Describe anticipated methods for assessing risk of bias of individual studies, including whether this will be done at the outcome or study level, or both; state how this information will be used in data synthesis	Page 9 , Lines 1-2
Data synthesis	15a	Describe criteria under which study data will be quantitatively synthesised	Page 9 , Lines 13-30<br>Page 10 , Lines 1-5
	15b	If data are appropriate for quantitative synthesis, describe planned summary measures, methods of handling data and methods of combining data from studies, including any planned exploration of consistency (such as I^2, Kendall's τ)	Page 9 , Lines 13-22
	15c	Describe any proposed additional analyses (such as sensitivity or subgroup analyses, meta-regression)	Page 10 , Lines 6-13
	15d	If quantitative synthesis is not appropriate, describe the type of summary planned	Page 9 , Lines 13-30<br>Page 10 , Lines 1-5
Meta-bias(es)	16	Specify any planned assessment of meta-bias(es) (such as publication bias across studies, selective reporting within studies)	Page 10 , Lines 14-16
Confidence in cumulative evidence	17	Describe how the strength of the body of evidence will be assessed (such as GRADE)	Page 9 , Lines 7-12

*** It is strongly recommended that this checklist be read in conjunction with the PRISMA-P Explanation and Elaboration (cite when available) for important clarification on the items. Amendments to a review protocol should be tracked and dated.** **The copyright for PRISMA-P (including checklist) is held by the PRISMA-P Group and is distributed under a Creative Commons Attribution Licence 4.0.**

From: Shamseer L, Moher D, Clarke M, Ghersi D, Liberati A, Petticrew M, Shekelle P, Stewart L, PRISMA-P Group. Preferred reporting items for systematic review and meta-analysis protocols (PRISMA-P) 2015: elaboration and explanation. BMJ. 2015 Jan 2;349(jan02 1):g7647.

For peer review only

BMJ Open

Nonpharmaceutical interventions in complementary and alternative medicine for insomnia in breast cancer survivors: a protocol for a systematic review and network meta-analysis

Journal:	*BMJ Open*
Manuscript ID	bmjopen-2023-071784.R1
Article Type:	Protocol
Date Submitted by the Author:	04-Apr-2023
Complete List of Authors:	Jin, Qi; Shanghai Municipal Hospital of Traditional Chinese Medicine<br>LIU, LUMIN; Long Hua Hospital,<br>Chen, Yue-lai; Long Hua Hospital<br>Yin, Ping; Long Hua Hospital
**Primary Subject Heading**:	Complementary medicine
Secondary Subject Heading:	Complementary medicine
Keywords:	COMPLEMENTARY MEDICINE, Quality in health care < HEALTH SERVICES ADMINISTRATION & MANAGEMENT, Breast tumours < ONCOLOGY

SCHOLARONE™ Manuscripts

Title Page

Title： Nonpharmaceutical interventions in complementary and alternative medicine for insomnia in breast cancer survivors: a protocol for a systematic review and network meta-analysis

Author information：

The full names of authors: Qi Jin, Lumin Liu, Yuelai Chen, Ping Yin

The affiliation(s) and address(es) of the author(s):

Qi Jin, Shanghai Municipal Hospital of Traditional Chinese Medicine, Shanghai University of Traditional Chinese Medicine, Shanghai 200071, China.

Email: jessyqi0606@163.com.

Lumin Liu, LongHua Hospital Shanghai University of Traditional Chinese Medicine, Shanghai 200032, China.

Email: beefllm@163.com.

Yuelai Chen, LongHua Hospital Shanghai University of Traditional Chinese Medicine, Shanghai 200032, China.

Email: chenyuelai@163.com.

Ping Yin, LongHua Hospital Shanghai University of Traditional Chinese Medicine, Shanghai 200032, China.

Email: bingxue616@163.com.

Co-first authors:

Qi Jin and Lumin Liu contributed equally to this article.

Corresponding author:

Dr. *Ping Yin*, LongHua Hospital Shanghai University of Traditional Chinese Medicine, Shanghai 200032, China. Email: bingxue616@163.com.

Professor *Yuelai Chen*, LongHua Hospital Shanghai University of Traditional Chinese Medicine, Shanghai 200032, China. Email: chenyuelai@163.com.

For peer review only - http://bmjopen.bmj.com/site/about/guidelines.xhtml

Nonpharmaceutical interventions in complementary and alternative medicine for insomnia in breast cancer survivors: a protocol for a systematic review and network meta-analysis

Qi Jin[2], Lumin Liu[1], Yuelai Chen[1], Ping Yin[1]

Author affiliations

[1.] LongHua Hospital Shanghai University of Traditional Chinese Medicine, Shanghai, China.

[2.] Shanghai Municipal Hospital of Traditional Chinese Medicine, Shanghai University of Traditional Chinese Medicine, Shanghai, China.

ABSTRACT

Introduction Insomnia has a high incidence in breast cancer patients, which not only affects the quality of life of patients, but also affects the efficiency of later treatment and rehabilitation. Although the sedative and hypnotic drugs commonly used in clinical practice have a rapid onset of action, they are also accompanied by different degrees of sequelae, withdrawal effects and dependence and addiction. Complementary and alternative medicine (CAM) or complementary and integrative medicine, complementary integrative therapies, including natural nutritional supplement therapy, psychotherapy, physical and mental exercise, physiotherapy have been reported to be used to treat cancer-related insomnia. Its clinical results are increasingly recognized and accepted by patients. However, the effectiveness and safety of these CAM are uneven, and there is no standard clinical application method. Therefore, in order to objectively evaluate the effects of different CAM on insomnia, we will conduct a network meta-analysis to explore the effects of different CAM interventions on improving sleep quality in breast cancer patients.

Methods and analysis We will search all Chinese and English databases from the inception of the database to 31 December, 2022. The databases include PubMed, Medline, Embase, Web of Science and Cochrane Central Register of Controlled Trials,

For peer review only - http://bmjopen.bmj.com/site/about/guidelines.xhtml

and the Chinese literature databases include CBM, CNKI, VIP, WANFANG. The insomnia severity index (ISI) and the Pittsburgh sleep quality index (PSQI) will be considered as the primary outcomes in the study. STATA V.15.0 software will be used to conduct pairwise meta-analysis and NMA. Finally, we will use the recommended risk assessment tool ROB2 for risk and bias assessment, and use the GRADE grading evaluation method to evaluate the quality of evidence.

Ethics and dissemination Ethical approval will not be required because the study will not include the original information of participants. The results will be published in a peer-reviewed journal or disseminated in relevant conferences.

Strengths and limitations of this study

► This study will compare the efficacy of complementary and alternative medicine (CAM) for insomnia in breast cancer survivors using network meta-analysis.

► The quality of evidence will be assessed by the Grading of Recommendations Assessment, Development and Evaluation system (GRADE) and the risk and bias assessment will be done by Risk of bias tool (ROB2).

► Participants who have received and are receiving chemotherapy, radiation, or surgery and participants who have never received chemotherapy, radiation, or surgery as covariates.

► We will only retrieve data from databases in Mandarin and English, which may restrict the amount of data available or lead to language bias.

PROSPERO registration number CRD42022382602.

INTRODUCTION

Insomnia, as one of the most prevalent complaints among cancer survivors, mainly manifests as difficulty in initiating and/or maintaining sleep, early morning awakening, which are associated with impaired performance and daytime well being.[1] According to the epidemiological investigation, 36%-59% patients experienced insomnia during the cancer trajectories. Among them, rates were greater in patients with breast cancer from 42% to 69%.[2] In a 5-year follow-up investigation, 38% disease-free breast cancer survivors reported that they still had sleep problems.[3] Insomnia in breast cancer

For peer review only - http://bmjopen.bmj.com/site/about/guidelines.xhtml

survivors can be debilitating, resulting in adverse impact on patients' life quality, and exacerbation on other symptoms, including pain, fatigue, hot flash and mental disorders.[4 5] Moreover, insomnia was associated with poor survival rate for patients with breast cancer.[6 7]

Currently the interventions in treating breast cancer related insomnia include pharmaceutical therapy and non-pharmaceutical ones. Due to the drug adverse effects, as well as the psychological and physical dependence, complementary and alternative medicine (CAM) has begun to receive more and more attention. According to the definition of CAM by NICCH, it is also called CIM (complementary and integrative medicine) or CIT (complementary integrative therapies), which includes four categories of treatment methods - natural nutrition supplements(such as dietary supplements, herbs and probiotics) , psychology (such as mindfulness, meditation)) , physical therapy (such as massage, spinal massage), a combination of psychosomatic exercises (such as yoga, tai chi, qigong, acupuncture) or psychological and nutritional (such as mindfulness diet) . [8] Cancer patients often use CAM therapy with recent estimates as high as 69%.[9] Most CAM methods, such as yoga, tai chi, meditation and music therapy, are safe for most patients. However, the use of herbs and supplements to treat sleep disturbance requires the supervision of health care professionals to prevent adverse reactions, interactions and toxicity.[10-12] Our network meta-analysis fully considered the compliance and safety of CAM in the treatment of insomnia in patients with breast cancer. Therefore, only non-drug intervention methods in CAM were retrieved, including psychotherapy (Cognitive-behavioral therapy for insomnia (CBT-I), mindfulness, psychological education guidance), psychosomatic exercise (yoga, tai chi, qigong, acupuncture) and different intensity physical exercise. The purpose is to compare the differences and safety of non-drug intervention in CAM in the treatment of insomnia in breast cancer, and offer some guidance and reference in clinical treatment.

Cognitive behavioral therapy for insomnia (CBT-I) has been demonstrated as an effective treatment and widely recommended for breast cancer survivors.[13 14] Evidence shows that acupuncture can improve sleep quality with mental and emotional distress

For peer review only - http://bmjopen.bmj.com/site/about/guidelines.xhtml

symptoms.[15 16] Mind-body therapies including yoga, taichi or qigong also seem to be available for breast cancer related insomnia.[17 18] Besides, several studies reported that physical exercise can relieve the insomnia disorder of breast cancer patients.[19 20] In conclusion, previous pairwise meta-analysis focused on only one CAM intervention mentioned above has demonstrated the efficacy in breast cancer related to insomnia.

However, apparent uncertainty about which non-pharmaceutical intervention is the most effective option makes it difficult for clinicians to manage this troublesome situation. In a randomized controlled trail, CBT-I showed superiority in shortening sleep onset latency, reducing wake, improving sleep efficiency while acupuncture was better at increasing total sleep time. Besides, the differences between acupuncture and CBT-I for women, non-whites, lower education, and those with pain were insignificant under subgroup analysis.[21] Thus, a comparison of these non-pharmaceutical interventions and the relative effectiveness between them represents an impending need for patients with breast cancer.

Network meta-analysis (NMA), which is also named multiple treatments comparison meta-analysis, helps to combine the direct and indirect evidence across a network of treatments. Furthermore, NMA is able to provide the ranking of treatment options based on their effectiveness.[22] However, to the best of our knowledge, so far NMA hasn't been used to perform the above functions in treating breast cancer related insomnia. Therefore, by the mean of systematic review (SR) and NMA, this study aims to compare the efficacy and evaluate the safety of different nonpharmaceutical CAM interventions including CBT-I, acupuncture, yoga, Tai chi, Qigong, mindfulness therapy, physical exercise of different intensities (aerobic and resistance), tuina, and massage for breast cancer survivors with insomnia.

In previous studies, there was only one SR report on physical exercise and mind-body exercise in the treatment of insomnia in breast cancer, and there was no specific SR study on insomnia in breast cancer patients for other CAM therapies.[17] Based on our knowledge, this may be the first SR and NMA study to compare the efficacy and safety of non-pharmaceutical complementary and alternative therapies in breast cancer insomnia survivors according to ISI, PSQI, and sleep diary related measures. The

For peer review only - http://bmjopen.bmj.com/site/about/guidelines.xhtml

results of the study will provide a ranking of different complementary alternative therapy interventions to assist patients, physicians, and policy makers in their decision making.

METHODS

Study registration

This protocol was registered on PROSPERO (CRD42022382602) and was reported following the Preferred Reporting Items for Systematic Review and Meta-Analysis Protocols (PRISMA-P) statement guidelines (online supplemental file 1 for PRISMA-P checklist). The findings of this study will be presented following the checklist of items to include when reporting a systematic review involving a network meta-analysis (PRISMA-NMA)

Inclusion criteria

Types of studies

We will incorporate RCTs that include multiple and single branches designs, full-text journal publications, unpublished clinical trials with online results in Chinese and English, and RCTs conducted in any healthcare setting.

Participants

Studies enrolled participants (18 years and older) who were diagnosed with breast cancer (stage 0-IV) and whose insomnia occurred after the breast cancer diagnosis. Patients who are receiving or have completed radiotherapy or chemotherapy or surgery and patients with breast cancer who have never received radiotherapy or chemotherapy or surgery. There will be no restrictions on race, nationality, and education.

Types of interventions

Nonpharmaceutical CAM interventions, as the main treatment, will be included, which will be limited to several aspects of the following four treatments. (1)Psychological: CBT-I with at least 2 components of cognitive therapy, stimulus control, sleep hygiene (including sleep education), sleep restriction, relaxation training, and other variants of CBT-I; Mindfulness, Meditation； (2) Physical: massage, tuina,

For peer review only - http://bmjopen.bmj.com/site/about/guidelines.xhtml

aerobic exercise, resistance exercise, walking, and interventions increasing physical activities; (3) Mind-body exercise (such as acupuncture, including acupuncture, electroacupuncture, auricular acupuncture, yoga, tai chi, Qigong). Studies used single or multiple complementary alternative medical intervention(s) will be considered.

Types of comparator(s)/control

The comparison group will be included usual care, a waiting control, placebo, sham acupuncture and sleep education.

Primary outcomes

The insomnia severity index (ISI) and the Pittsburgh sleep quality index (PSQI) will be considered as the primary outcomes in the study.

The ISI consists of 7 items to assess the nature and symptoms of the subject's sleep disorders. Questions address subjects' subjective ratings of sleep quality, including the severity of symptoms, subjects' satisfaction with their sleep patterns, the impact of the degree of insomnia on daily functioning, subjects' awareness of the impact of insomnia on themselves, and their level of frustration due to their sleep disorders.[23]

PSQI instrument produces a global sleep quality score and seven specific component scores: quality, latency, duration, disturbance, habitual sleep efficiency, use of sleeping medications, and daytime dysfunction. Global scores, ranging from 0 to 21 with higher scores, indicates poor sleep quality and high sleep disturbance.[24]

Secondary outcomes

Sleep efficiency, sleep latency (SOL), wake-up time after sleep onset (WASO), and total sleep time (TST) derived from polysomnography data, Actigraphy data or sleep diaries will be regarded as secondary outcome measures. In addition, quantitative statistics of adverse events as another secondary indicator.[25]

Exclusion criteria

1. Studies that are duplicates.
2. Patients with other diseases that affect insomnia.
3. The design type is non- RCT, including conference paper, summary, abstracts, editorials, clinical observations, case studies, cohort studies, non-randomized trials,

For peer review only - http://bmjopen.bmj.com/site/about/guidelines.xhtml

case-control studies, any non-experimental investigation (including cross-sectional and retrospective surveys).

4. We generally exclude studies involving various types of cancer unless they have subgroup data for breast cancer.

5. The full text of the study is not available.

Any study that meets one or more of the above criteria will not be considered.

Search methods for identification of studies

We will search all Chinese and English databases from the inception of the database to 31 December, 2022. The English literature databases include PubMed, Medline, Embase, Web of Science and Cochrane Central Register of Controlled Trials, and the Chinese literature databases include CBM, CNKI, VIP, WANFANG. The search keywords were focused on 'CAM' 'CIM', 'CIT', ' breast cancer ', ' insomnia ', ' acupuncture ', ' electroacupuncture ', ' Auricular acupuncture ', ' cognitive behavioral therapy ', ' Mind-body therapies ', 'mindfulness', 'meditation', 'tuina', 'masssage',' yoga ', ' tai chi ', ' qigong ', ' physical exercise ', ' resistance training ',' Walking ',' randomized controlled trial '. The final search formula was obtained by searching for the subject words, subordinate words and free words. The reference list of all selected articles will be independently screened to identify additional studies missed in the initial search. Table 1 shows specific search strategies for Web of science.

Table 1 Search strategy for the Web of Science

Number	Search items
**1**	TS=(breast neoplasms OR breast cancer OR breast tumor OR mammary cancer OR breast carcinoma)
**2**	TS=(sleep initiation and maintenance disorders OR insomnia)
**3**	TS=(complementary therapies OR complementary medicine OR alternative medicine OR alternative therapies OR integrative medicine
**4**	TS=(acupuncture OR electroacupuncture OR auricular acupuncture OR tuina OR massage)
**5**	TS=(cognitive behavioral therapy OR mindfulness OR meditation)
**6**	TS=(mind-body therapies)
**7**	TS=(yoga)
**8**	TS=(tai ji OR tai chi)
**9**	TS=(qigong)

For peer review only - http://bmjopen.bmj.com/site/about/guidelines.xhtml

**10**	TS=(exercise therapy OR physical exercise OR aerobic exercise)
**11**	TS=(resistance training)
**12**	TS=(walking)
**13**	TS=(randomized controlled trial OR controlled clinical trial OR randomized OR placebo OR clinical trials as topic OR randomly OR trial)
**14**	TS=(animals)
**15**	TS=(humans AND animals)
**16**	((#13) NOT #14) NOT #15
**17**	#3 OR #4 OR #5 OR #6 OR #7 OR #8 OR #9 OR #10 OR #11 OR #12
**18**	#1 AND #2 AND #16 AND #17

Data collection and analysis

Selection of studies

Two researchers (QJ and LML) will screen the literature independently and extract the data and cross-check them. If there are differences, these will be resolved through discussion or negotiation with third parties. In the literature screening, the title of the article will be first read. After excluding obviously unrelated literatures, the abstract and full text will be further read to determine whether it will be included. If necessary, the authors of the original studies will be contacted by email or telephone to obtain information that has not been identified but is important to this study. EndnoteX9 will be used to manage retrieved studies and remove duplicates. The selection procedure is presented in a PRISMA flow chart (Figure 1).

Data extraction and management

Two researchers (QJ and LML) will independently extract data based on a predesigned form. The extracted data will be as follows:(1) Basic information included in the study: first author and year of publication; (2) Research methods: research type, intervention measures, control measures, sample size; (3) Concerned Outcome indicators and outcome measurement data. (4) Follow-up. The corresponding authors will be contacted for missing information. The two researchers will crosscheck the data after completion of data extraction. The disagreements will be solved by the team discussion or consultation with the third researcher (YLC).

Quality assessment

Two independent reviewers (QJ and PY) will complete the risk-of-bias assessment

For peer review only - http://bmjopen.bmj.com/site/about/guidelines.xhtml

and evaluation of evidence certainty.

We will use Version 2 of the Cochrane tool for assessing the risk of bias in randomized trials (RoB2) revised by the Cochrane Methodology Group in 2019.[26] The quality of the study will be evaluated in the following five areas: (1) bias in the randomization process, (2) deviation from established interventions, (3) bias in missing outcome data, (4) bias in outcome measurement, and (5) selective reporting of bias in results.

The Grading of Recommendations Assessment, Development and Evaluation System (GRADE) system will be used to grade the quality of the evidence for main outcomes.[27] Evidence quality will be rated 'high', 'moderate', 'low' or 'very low' according to the GRADE rating standards. The evidence quality of a specific study will be assessed according to the risk of bias, inconsistency, indirectness, imprecision and publication bias.

Pairwise meta-analysis

The pairwise meta-analysis will be performed using STATA 15.0. Conventional pairwise meta-analyses will be performed with random-effects model. Pooled odds ratio (OR) with 95% confidence interval (CI) will be used for the dichotomous variables. Standardized mean differences (SMDs) or weighted mean differences (WMDs) with 95% CI will be used for the continuous variables. The heterogeneity between trials will be estimated using I^2 statistics and P value. The values of 25%, 50%, and 75% for the I^2 as indicative of low, moderate, and high statistical heterogeneity, respectively. We will explore sources of heterogeneity by subgroup analysis and meta-regression.[28]

Network meta-analysis

If enough data is available, we will conduct a network meta-analysis (NMA) to compare the efficacy of different interventions and controls. The NMA will be conducted on both direct evidence and indirect evidence in a Bayesian framework using STATA V.15.0 software. The publication bias will be evaluated with the Egger's test and funnel plots if the number of studies exceeds. A 2-tailed P value < 0.05 is considered as statistically significant.[29] In addition, a node-splitting model will also be used to assess local inconsistency at the network level, and a consistency or

For peer review only - http://bmjopen.bmj.com/site/about/guidelines.xhtml

inconsistency model will be selected based on the results. If $P<0.05$, there was a statistically significant difference between the indirect and direct multiple treatment comparisons. To evaluate the convergence of the results, we will analyze the potential scale reduction factor (PSRF), where a PSRF value close to 1 indicates successful convergence.[30]

Subgroup analysis, meta regression analysis and sensitivity analysis

Subgroup analysis and meta-regression analysis will be used to explore possible sources of heterogeneity and inconsistency. Subgroup analysis will be performed according to the different types of non-drug interventions. We will conduct a meta-regression analysis with participants who have received or are receiving or have never received chemotherapy, radiation, or surgery as covariates. Sensitivity analysis is carried out by the method of exclusion to verify the robustness of the results.

Publication bias assessment

If more than 10 studies are included, a comparison-adjusted funnel plot will be generated to detect reporting bias.

Patients and public involvement

This is a SR and NMA based on existing studies, so no patients or members of the public will directly be involved.

Ethics and dissemination

The study will not require ethical approval because it comprises analysis based on existing studies. The results are expected to be published in a peer-reviewed journal or disseminated at relevant conferences. The objective of our study will evaluate the impact of complementary alternative therapies (including acupuncture, CBT-I, physical exercise, mind-body exercise etc.) on sleep-related outcomes in breast cancer survivors with insomnia by conducting a systematic review and network meta-analysis of existing randomized controlled trial data for the purpose of providing reliable evidence-based medical evidence for complementary and alternative therapies for insomnia in breast cancer.

Contributors

For peer review only - http://bmjopen.bmj.com/site/about/guidelines.xhtml

QJ and LML contributed equally to this article as co-first authors. YLC and PY contributed to the design of the study. The manuscript was drafted by QJ and LML. QJ and LML participated in the design of the search strategy and data extraction dataset. YLC and PY will monitor each procedure of the review and be responsible for quality control. All the authors participated in the reading, discussion and revision of the manuscript, and all approved of the protocol for publication.

Funding

This work was supported by the Clinical Incubation Program of the National Medical Center of LongHua Hospital to Shanghai University of Traditional Chinese Medicine, with grant number (GY202201).

Competing interests

None declared.

Patient and public involvement

Patients and/or the public were not involved in the design, conduct, reporting, or dissemination plans of this research.

Patient consent for publication

Not applicable.

Provenance and peer review

Not commissioned; externally peer reviewed.

For peer review only - http://bmjopen.bmj.com/site/about/guidelines.xhtml

Reference

1. Schieber K, Niecke A, Geiser F, *et al*. The course of cancer-related insomnia: don't expect it to disappear after cancer treatment. *Sleep medicine* 2019;58:107-13.
2. Savard J, Ivers H, Villa J, *et al*. Natural course of insomnia comorbid with cancer: an 18-month longitudinal study. *Journal of clinical oncology: official journal of the American Society of Clinical Oncology* 2011;29(26):3580-6.
3. Schmidt ME, Wiskemann J, Steindorf K. Quality of life, problems, and needs of disease-free breast cancer survivors 5 years after diagnosis. *Quality of life research: an international journal of quality of life aspects of treatment, care and rehabilitation* 2018;27(8):2077-86.
4. Collins KP, Geller DA, Antoni M, *et al*. Sleep duration is associated with survival in advanced cancer patients. *Sleep medicine* 2017;32:208-12.
5. Narayanan S, Reddy A, Lopez G, *et al*. Sleep disturbance in cancer patients referred to an ambulatory integrative oncology consultation. *Supportive care in cancer: official journal of the Multinational Association of Supportive Care in Cancer* 2022;30(3):2417-25.
6. Costa AR, Fontes F, Pereira S, *et al*. Impact of breast cancer treatments on sleep disturbances - A systematic review. *Breast (Edinburgh, Scotland)* 2014;23(6):697-709.
7. Savard J, Ivers H, Savard MH, *et al*. Cancer treatments and their side effects are associated with aggravation of insomnia: Results of a longitudinal study. *Cancer* 2015;121(10):1703-11.
8. National Center for Complementary and Integrative Health (NCCIH) (2016) Complementary, alternative, or integrative health: what's in a name? Secondary National Center for Complementary and Integrative Health (NCCIH) (2016) Complementary, alternative, or integrative health: what's in a name? https://nccih.nih.gov/health/integrative-health#integrative.
9. Smith JA. Assessment of the Utilization of Complimentary and Alternative Medication in Women with Gynecologic or Breast Cancers. 2015.
10. White CM. Dietary Supplements Pose Real Dangers to Patients. *The Annals of pharmacotherapy* 2020;54(8):815-19.
11. Shinjyo N, Waddell G, Green J. Valerian Root in Treating Sleep Problems and Associated Disorders-A Systematic Review and Meta-Analysis. *Journal of evidence-based integrative medicine* 2020;25:2515690x20967323.
12. Richardson MA, Sanders T, Palmer JL, *et al*. Complementary/alternative medicine use in a comprehensive cancer center and the implications for oncology. *Journal of clinical oncology : official journal of the American Society of Clinical Oncology* 2000;18(13):2505-14.
13. Ma Y, Hall DL, Ngo LH, *et al*. Efficacy of cognitive behavioral therapy for insomnia in breast cancer: A meta-analysis. *Sleep medicine reviews* 2021;55:101376.
14. Bean HR, Diggens J, Ftanou M, *et al*. Light enhanced cognitive behavioral therapy for insomnia and fatigue during chemotherapy for breast cancer: a randomized controlled trial. *Sleep* 2022;45(3).
15. Zhang J, Zhang Z, Huang S, *et al*. Acupuncture for cancer-related insomnia: A systematic review and meta-analysis. *Phytomedicin: international journal of phytotherapy and phytopharmacology* 2022;102:154160.
16. Garland SN, Xie SX, Li Q, *et al*. Comparative effectiveness of electro-acupuncture versus

For peer review only - http://bmjopen.bmj.com/site/about/guidelines.xhtml

gabapentin for sleep disturbances in breast cancer survivors with hot flashes: a randomized trial. *Menopause (New York, NY)* 2017;24(5):517-23.

17. Kreutz C, Schmidt ME, Steindorf K. Effects of physical and mind-body exercise on sleep problems during and after breast cancer treatment: a systematic review and meta-analysis. *Breast cancer research and treatment* 2019;176(1):1-15.
18. Chaoul A, Milbury K, Spelman A, *et al*. Randomized trial of Tibetan yoga in patients with breast cancer undergoing chemotherapy. *Cancer* 2018;124(1):36-45.
19. Mercier J, Savard J, Bernard P. Exercise interventions to improve sleep in cancer patients: A systematic review and meta-analysis. *Sleep medicine reviews* 2017;36:43-56.
20. Steindorf K, Wiskemann J, Ulrich CM, *et al*. Effects of exercise on sleep problems in breast cancer patients receiving radiotherapy: a randomized clinical trial. *Breast cancer research and treatment* 2017;162(3):489-99.
21. Garland SN, Xie SX, DuHamel K, *et al*. Acupuncture Versus Cognitive Behavioral Therapy for Insomnia in Cancer Survivors: A Randomized Clinical Trial. *Journal of the National Cancer Institute* 2019;111(12):1323-31.
22. Bafeta A, Trinquart L, Seror R, *et al.* Reporting of results from network meta-analyses: methodological systematic review. *BMJ (Clinical research ed)* 2014;348:g1741.
23. Morin CM, Belleville G, Bélanger L, *et al*. The Insomnia Severity Index: psychometric indicators to detect insomnia cases and evaluate treatment response. *Sleep* 2011;34(5):601-8.
24. Buysse DJ, Reynolds CF, 3rd, Monk TH, *et al.* The Pittsburgh Sleep Quality Index: a new instrument for psychiatric practice and research. *Psychiatry research* 1989;28(2):193-213.
25. Fekedulegn D, Andrew ME, Shi M, *et al.* Actigraphy-Based Assessment of Sleep Parameters. *Annals of work exposures and health* 2020;64(4):350-67.
26. Sterne JAC, Savović J, Page MJ, *et al*. RoB 2: a revised tool for assessing risk of bias in randomised trials. *BMJ (Clinical research ed)* 2019;366:l4898.
27. Guyatt GH, Oxman AD, Vist GE, *et al.* GRADE: an emerging consensus on rating quality of evidence and strength of recommendations. *BMJ (Clinical research ed)* 2008;336(7650):924-6.
28. Higgins JP, Thompson SG, Deeks JJ, *et al.* Measuring inconsistency in meta-analyses. *BMJ (Clinical research ed)* 2003;327(7414):557-60.
29. Salanti G, Ades AE, Ioannidis JP. Graphical methods and numerical summaries for presenting results from multiple-treatment meta-analysis: an overview and tutorial. *Journal of clinical epidemiology* 2011;64(2):163-71.
30. BROOKS, Stephen P, GELMAN, *et al.* General methods for monitoring convergence of iterative simulations. 1998.

Figure Legend

Figure 1 PRISMA flow diagram of the study selection process

Table

Table 1 Search strategy for the Web of Science

For peer review only - http://bmjopen.bmj.com/site/about/guidelines.xhtml

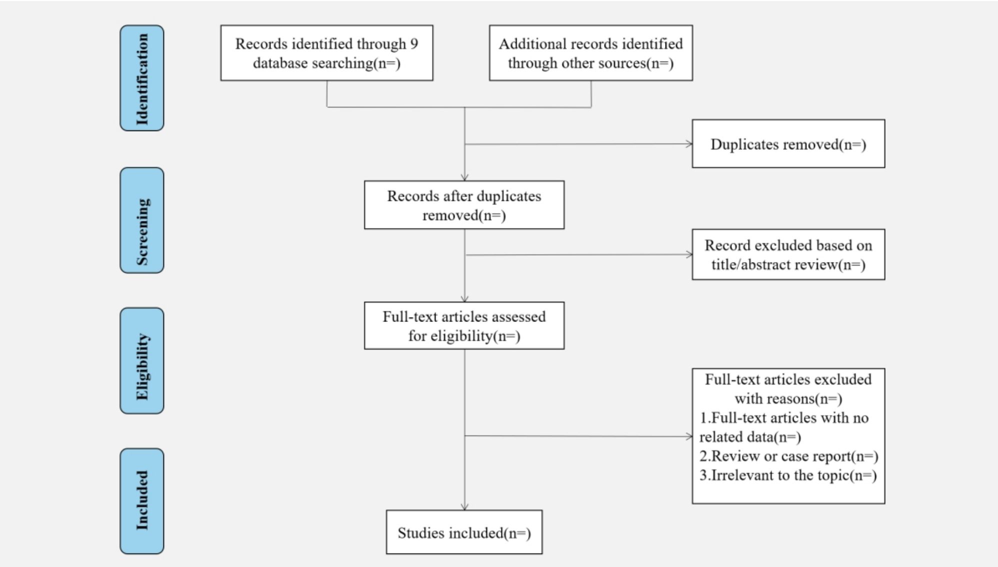

Figure 1 PRISMA flow diagram of the study selection process

276x167mm (192 x 192 DPI)

Search strategies

Pubmed:

(((("breast neoplasms"[Mesh]) OR (breast cancer[tiab]) OR (breast tumor[tiab]) OR (mammary cancer[tiab]) OR (breast carcinoma[tiab])) AND (("sleep initiation and maintenance disorders"[Mesh]) OR (insomnia[tiab]))) AND ((("complementary therapies"[Mesh]) OR (complementary medicine[tiab]) OR (alternative medicine[tiab]) OR (alternative therapies[tiab]) OR (integrative medicine[tiab])) OR (("acupuncture"[Mesh]) OR (electroacupuncture[tiab]) OR (auricular acupuncture[tiab])) OR ("cognitive behavioral therapy"[Mesh]) OR ("massage"[Mesh]) OR (tuina[tiab]) OR ("mindfulness"[Mesh]) OR ("meditation"[Mesh]) OR ("mind-body therapies"[Mesh]) OR ("yoga"[Mesh]) OR (("tai ji"[Mesh]) OR (tai chi[tiab])) OR ("qigong"[Mesh]) OR (("exercise"[Mesh]) OR (physical exercise[tiab]) OR (aerobic exercise[tiab])) OR ("resistance training"[Mesh]) OR ("walking"[Mesh]))) AND ((randomized controlled trial[pt] OR controlled clinical trial[pt] OR randomized[tiab] OR placebo[tiab] OR randomly[tiab] OR trial[tiab] OR groups[tiab]) NOT (animals[mh] NOT humans[mh]))

Web of science:

Table 1 Search strategy for the Web of Science

Number	Search items
1	TS=(breast neoplasms OR breast cancer OR breast tumor OR mammary cancer OR breast carcinoma)
2	TS=(sleep initiation and maintenance disorders OR insomnia)
3	TS=(complementary therapies OR complementary medicine OR alternative medicine OR alternative therapies OR integrative medicine
4	TS=(acupuncture OR electroacupuncture OR auricular acupuncture OR tuina OR massage)
5	TS=(cognitive behavioral therapy OR mindfulness OR meditation)
6	TS=(mind-body therapies)
7	TS=(yoga)

For peer review only - http://bmjopen.bmj.com/site/about/guidelines.xhtml

**8**	TS=(tai ji OR tai chi)
**9**	TS=(qigong)
**10**	TS=(exercise therapy OR physical exercise OR aerobic exercise)
**11**	TS=(resistance training)
**12**	TS=(walking)
**13**	TS=(randomized controlled trial OR controlled clinical trial OR randomized OR placebo OR clinical trials as topic OR randomly OR trial)
**14**	TS=(animals)
**15**	TS=(humans AND animals)
**16**	((#13) NOT #14) NOT #15
**17**	#3 OR #4 OR #5 OR #6 OR #7 OR #8 OR #9 OR #10 OR #11 OR #12
**18**	#1 AND #2 AND #16 AND #17

Medline:

1. breast neoplasms[Mesh] or (breast cancer or breast tumor or mammary cancer or breast carcinoma).ti,ab.

2. (sleep initiation and maintenance disorders).mp. or (insomnia).ti,ab.

3. complementary therapies[Mesh] or (complementary medicine or alternative medicine or alternative therapies or integrative medicine).ti,ab.

4. acupuncture[Mesh] or (electroacupuncture or auricular acupuncture).ti,ab.

5. massage[Mesh] or (tuina).ti,ab.

6. cognitive behavioral therapy[Mesh]

7. mindfulness[Mesh]

8. meditation[Mesh]

9. mind-body therapies[Mesh]

10. yoga[Mesh]

11. tai ji[Mesh] or (tai chi).ti,ab.

12. qigong[Mesh]

13. exercise[Mesh] or (physical exercise or aerobic exercise).ti,ab.

14. resistance training[Mesh]

For peer review only

15. walking[Mesh]

16. (randomized controlled trial or controlled clinical trial).pt. or random$.ab. or trial.ab.

17. 3 or 4 or 5 or 6 or 7 or 8 or 9 or 10 or 11 or 12 or 13 or 14 or 15

18. 1 and 2 and 16 and 17

Embase:

1. 'breast neoplasms'/exp OR 'breast neoplasms' OR 'breast cancer':ab,ti OR 'breast tumor':ab,ti OR 'mammary cancer':ab,ti OR 'breast carcinoma':ab,ti

2. 'sleep initiation and maintenance disorder'/exp OR 'sleep initiation and maintenance disorder' OR 'insomnia':ab,ti

3. 'complementary therapies'/exp OR 'complementary therapies' OR 'complementary medicine':ab,ti OR 'alternative medicine':ab,ti OR 'alternative therapies':ab,ti OR 'integrative medicine':ab,ti

4. 'acupuncture'/exp OR 'acupuncture' OR 'electroacupuncture':ab,ti OR 'auricular acupuncture':ab,ti

5. 'massage'/exp OR 'massage' OR 'tuina':ab,ti

6. 'cognitive behavioral therapy'/exp OR 'cognitive behavioral therapy'

7. 'mindfulness'/exp OR 'mindfulness'

8. 'meditation'/exp OR 'meditation'

9. 'mind-body therapies'/exp OR 'mind-body therapies'

10. 'yoga'/exp OR 'yoga'

11. 'tai ji'/exp OR 'tai ji' OR 'tai chi':ab,ti

12. 'qigong'/exp OR 'qigong'

13. 'exercise'/exp OR 'exercise' OR 'physical exercise':ab,ti OR 'aerobic exercise':ab,ti

14. 'resistance training'/exp OR 'resistance training'

15. 'walking'/exp OR 'walking'

16. 'crossover procedure':de OR 'double-blind procedure':de OR 'randomized controlled trial':de OR 'single-blind procedure':de OR (random* OR factorial* OR crossover* OR cross NEXT/1 over* OR placebo* OR doubl* NEAR/1 blind* OR singl* NEAR/1 blind* OR assign* OR allocat* OR volunteer*):de,ab,ti

For peer review only

17. 3 or 4 or 5 or 6 or 7 or 8 or 9 or 10 or 11 or 12 or 13 or 14 or 15

18. 1 and 2 and 16 and 17

Cochrane:

#1 ((breast neoplasms) OR (breast cancer) OR (breast tumor) OR (mammary cancer) OR (breast carcinoma)):ab,ti,kw

#2 (sleep initiation and maintenance disorders) OR (insomnia):ab,ti,kw

#3 (((complementary therapies) OR (complementary medicine) OR (alternative medicine) OR (alternative therapies) OR (integrative medicine)) OR ((acupuncture) OR (electroacupuncture) OR (auricular acupuncture) OR (tuina) OR (massage)) OR ((cognitive behavioral therapy) OR (mindfulness) OR (meditation)) OR (mind-body therapies) OR (yoga) OR ((tai ji) OR (tai chi)) OR (qigong) OR ((exercise therapy) OR (physical exercise) OR (aerobic exercise)) OR (resistance training) OR (walking)):ab,ti,kw

#1 AND #2 AND #3

CBM:

("失眠"[摘要] OR "不寐"[摘要] OR "睡眠障碍"[摘要] OR "睡眠"[摘要]) AND ("乳腺癌"[摘要] OR "乳腺肿瘤"[摘要]) AND ("补充医学"[摘要] OR "替代医学"[摘要] OR "结合医学"[摘要] OR "针刺"[摘要] OR "针灸"[摘要] OR "电针"[摘要] OR "耳针"[摘要] OR "推拿"[摘要] OR "按摩"[摘要] OR "正念"[摘要] OR "冥想"[摘要] OR "认知行为"[摘要] OR "瑜伽"[摘要] OR "太极"[摘要] OR "气功"[摘要] OR "有氧"[摘要] OR "运动"[摘要] OR "体育锻炼"[摘要] OR "身心"[摘要] OR "抗阻"[摘要])

CNKI:

(TKA = '失眠' OR TKA = '不寐' OR TKA = '睡眠' OR TKA = '睡眠障碍') AND (TKA = '乳腺癌' OR TKA = '乳腺肿瘤') AND (TKA = '补充医学' OR TKA = '替代医学' OR TKA = '结合医学' OR TKA = '针刺' OR TKA = '针灸' OR TKA = '电针' OR TKA = '耳针' OR TKA = '推拿' OR TKA = '按摩' OR TKA = '正念' OR TKA = '冥想' OR TKA = '认知行为' OR TKA = '瑜伽' OR TKA = '太极' OR TKA = '气功'

OR TKA = '有氧' OR TKA = '运动' OR TKA = '体育锻炼' OR TKA = '身心' OR TKA = '抗阻')

VIP：

M=(失眠 OR 不寐 OR 睡眠 OR 睡眠障碍) AND M=(乳腺癌 OR 乳腺肿瘤) AND M=(补充医学 OR 替代医学 OR 结合医学 OR 针刺 OR 针灸 OR 电针 OR 耳针 OR 推拿 OR 按摩 OR 正念 OR 冥想 OR 认知行为 OR 瑜伽 OR 太极 OR 气功 OR 有氧 OR 运动 OR 体育锻炼 OR 身心 OR 抗阻)

WANFANG：

题名或关键词:(("失眠" or "不寐" or "睡眠" or "睡眠障碍") and ("乳腺癌" or "乳腺肿瘤") and ("补充医学" or "替代医学" or "结合医学" or "针刺" or "针灸" or "电针" or "耳针" or "推拿" or "按摩" or "正念" or "冥想" or "认知行为" or "瑜伽" or "太极" or "气功" or "有氧" or "运动" or "体育锻炼" or "身心" or "抗阻"))

For peer review only

PRISMA-P (Preferred Reporting Items for Systematic review and Meta-Analysis Protocols) 2015 checklist: recommended items to address in a systematic review protocol*

Section and topic	Item No	Checklist item	Location in the article
**ADMINISTRATIVE INFORMATION**			
Title:			
Identification	1a	Identify the report as a protocol of a systematic review	Page 1, Lines 2-3
Update	1b	If the protocol is for an update of a previous systematic review, identify as such	NA
Registration	2	If registered, provide the name of the registry (such as PROSPERO) and registration number	Page 4 , Line 28
Authors:			
Contact	3a	Provide name, institutional affiliation, e-mail address of all protocol authors; provide physical mailing address of corresponding author	Page 1 , Lines 6-29
Contributions	3b	Describe contributions of protocol authors and identify the guarantor of the review	Page 11 , Lines 29-30<br>Page 12 , Lines 1-5
Amendments	4	If the protocol represents an amendment of a previously completed or published protocol, identify as such and list changes; otherwise, state plan for documenting important protocol amendments	NA
Support:			
Sources	5a	Indicate sources of financial or other support for the review	Page 12 , Lines 6-9
Sponsor	5b	Provide name for the review funder and/or sponsor	Page 12 , Lines 6-9
Role of sponsor or funder	5c	Describe roles of funder(s), sponsor(s), and/or institution(s), if any, in developing the protocol	NA
**INTRODUCTION**			
Rationale	6	Describe the rationale for the review in the context of what is already known	Page 3 , Lines 12-30<br>Page 4 , Lines 1-21
Objectives	7	Provide an explicit statement of the question(s) the review will address with reference to participants, interventions, comparators, and outcomes (PICO)	Page 4 , Lines 22-24
**METHODS**			
Eligibility criteria	8	Specify the study characteristics (such as PICO, study design, setting, time frame) and report characteristics (such as years considered, language, publication status) to be used as criteria for eligibility for the review	Page 5 , Lines 5-29<br>Page 6 , Lines 1-28
Information sources	9	Describe all intended information sources (such as electronic databases, contact with study authors, trial registers or other grey literature sources) with planned dates of coverage	Page 7 , Lines 1-12

For peer review only

Search strategy	10	Present draft of search strategy to be used for at least one electronic database, including planned limits, such that it could be repeated	Page 7 , Table1
Study records:			
Data management	11a	Describe the mechanism(s) that will be used to manage records and data throughout the review	Page 8 , Line 10
Selection process	11b	State the process that will be used for selecting studies (such as two independent reviewers) through each phase of the review (that is, screening, eligibility and inclusion in meta-analysis)	Page 8 , Lines 3-9
Data collection process	11c	Describe planned method of extracting data from reports (such as piloting forms, done independently, in duplicate), any processes for obtaining and confirming data from investigators	Page 8 , Lines 18-20
Data items	12	List and define all variables for which data will be sought (such as PICO items, funding sources), any pre-planned data assumptions and simplifications	Page 8 , Lines 14-17
Outcomes and prioritization	13	List and define all outcomes for which data will be sought, including prioritization of main and additional outcomes, with rationale	Page 6 , Lines 1-17
Risk of bias in individual studies	14	Describe anticipated methods for assessing risk of bias of individual studies, including whether this will be done at the outcome or study level, or both; state how this information will be used in data synthesis	Page 9 , Lines 1-2
Data synthesis	15a	Describe criteria under which study data will be quantitatively synthesised	Page 9 , Lines 13-30<br>Page 10 , Lines 1-5
	15b	If data are appropriate for quantitative synthesis, describe planned summary measures, methods of handling data and methods of combining data from studies, including any planned exploration of consistency (such as I^2, Kendall's τ)	Page 9 , Lines 13-22
	15c	Describe any proposed additional analyses (such as sensitivity or subgroup analyses, meta-regression)	Page 10 , Lines 6-13
	15d	If quantitative synthesis is not appropriate, describe the type of summary planned	Page 9 , Lines 13-30<br>Page 10 , Lines 1-5
Meta-bias(es)	16	Specify any planned assessment of meta-bias(es) (such as publication bias across studies, selective reporting within studies)	Page 10 , Lines 14-16
Confidence in cumulative evidence	17	Describe how the strength of the body of evidence will be assessed (such as GRADE)	Page 9 , Lines 7-12

*** It is strongly recommended that this checklist be read in conjunction with the PRISMA-P Explanation and Elaboration (cite when available) for important clarification on the items. Amendments to a review protocol should be tracked and dated. The copyright for PRISMA-P (including checklist) is held by the PRISMA-P Group and is distributed under a Creative Commons Attribution Licence 4.0.**

From: Shamseer L, Moher D, Clarke M, Ghersi D, Liberati A, Petticrew M, Shekelle P, Stewart L, PRISMA-P Group. Preferred reporting items for systematic review and meta-analysis protocols (PRISMA-P) 2015: elaboration and explanation. BMJ. 2015 Jan 2;349(jan02 1):g7647.

For peer review only

BMJ Open

Nonpharmaceutical interventions in complementary and alternative medicine for insomnia in breast cancer survivors: a protocol for a systematic review and network meta-analysis

Journal:	*BMJ Open*
Manuscript ID	bmjopen-2023-071784.R2
Article Type:	Protocol
Date Submitted by the Author:	16-Apr-2023
Complete List of Authors:	Jin, Qi; Shanghai Municipal Hospital of Traditional Chinese Medicine<br>LIU, LUMIN; Long Hua Hospital,<br>Chen, Yue-lai; Long Hua Hospital<br>Yin, Ping; Long Hua Hospital
**Primary Subject Heading**:	Complementary medicine
Secondary Subject Heading:	Complementary medicine
Keywords:	COMPLEMENTARY MEDICINE, Quality in health care < HEALTH SERVICES ADMINISTRATION & MANAGEMENT, Breast tumours < ONCOLOGY

SCHOLARONE™
Manuscripts

Title Page

Title： Nonpharmaceutical interventions in complementary and alternative medicine for insomnia in breast cancer survivors: a protocol for a systematic review and network meta-analysis

Author information：

The full names of authors: Qi Jin, Lumin Liu, Yuelai Chen, Ping Yin

The affiliation(s) and address(es) of the author(s):

Qi Jin, Shanghai Municipal Hospital of Traditional Chinese Medicine, Shanghai University of Traditional Chinese Medicine, Shanghai 200071, China.

Email: jessyqi0606@163.com.

Lumin Liu, LongHua Hospital Shanghai University of Traditional Chinese Medicine, Shanghai 200032, China.

Email: beefllm@163.com.

Yuelai Chen, LongHua Hospital Shanghai University of Traditional Chinese Medicine, Shanghai 200032, China.

Email: chenyuelai@163.com.

Ping Yin, LongHua Hospital Shanghai University of Traditional Chinese Medicine, Shanghai 200032, China.

Email: bingxue616@163.com.

Co-first authors:

Qi Jin and Lumin Liu contributed equally to this article.

Corresponding author:

Dr. *Ping Yin*, LongHua Hospital Shanghai University of Traditional Chinese Medicine, Shanghai 200032, China. Email: bingxue616@163.com.

Professor *Yuelai Chen*, LongHua Hospital Shanghai University of Traditional Chinese Medicine, Shanghai 200032, China. Email: chenyuelai@163.com.

For peer review only

For peer review only - http://bmjopen.bmj.com/site/about/guidelines.xhtml

Nonpharmaceutical interventions in complementary and alternative medicine for insomnia in breast cancer survivors: a protocol for a systematic review and network meta-analysis

Qi Jin[2], Lumin Liu[1], Yuelai Chen[1], Ping Yin[1]

Author affiliations

[1.] LongHua Hospital Shanghai University of Traditional Chinese Medicine, Shanghai, China.

[2.] Shanghai Municipal Hospital of Traditional Chinese Medicine, Shanghai University of Traditional Chinese Medicine, Shanghai, China.

ABSTRACT

Introduction Insomnia has a high incidence in breast cancer patients, which not only affects the quality of life of patients, but also affects the efficiency of later treatment and rehabilitation. Although the sedative and hypnotic drugs commonly used in clinical practice have a rapid onset of action, they are also accompanied by different degrees of sequelae, withdrawal effects and dependence and addiction. Complementary and alternative medicine (CAM) or complementary and integrative medicine, complementary integrative therapies, including natural nutritional supplement therapy, psychotherapy, physical and mental exercise, physiotherapy have been reported to be used to treat cancer-related insomnia. Its clinical results are increasingly recognized and accepted by patients. However, the effectiveness and safety of these CAM are uneven, and there is no standard clinical application method. Therefore, in order to objectively evaluate the effects of different CAM on insomnia, we will conduct a network meta-analysis to explore the effects of different CAM interventions on improving sleep quality in breast cancer patients.

Methods and analysis We will search all Chinese and English databases from the inception of the database to 31 December, 2022. The databases include PubMed, Medline, Embase, Web of Science and Cochrane Central Register of Controlled Trials,

For peer review only - http://bmjopen.bmj.com/site/about/guidelines.xhtml

and the Chinese literature databases include CBM, CNKI, VIP, WANFANG. The insomnia severity index (ISI) and the Pittsburgh sleep quality index (PSQI) will be considered as the primary outcomes in the study. STATA V.15.0 software will be used to conduct pairwise meta-analysis and NMA. Finally, we will use the recommended risk assessment tool ROB2 for risk and bias assessment, and use the GRADE grading evaluation method to evaluate the quality of evidence.

Ethics and dissemination Ethical approval will not be required because the study will not include the original information of participants. The results will be published in a peer-reviewed journal or disseminated in relevant conferences.

Strengths and limitations of this study

► This study comprehensively compares the efficacy and safety of different non-pharmacological interventions for insomnia in breast cancer survivors to rank the superior efficacy of different interventions using network meta-analysis.

► The quality of evidence will be assessed by the Grading of Recommendations Assessment, Development and Evaluation system (GRADE) and the risk and bias assessment will be done by Risk of bias tool (ROB2).

► We will perform subgroup analyses based on different types of non-pharmacological interventions and conduct a meta-regression analysis with participants who have received or are receiving or have never received chemotherapy, radiation, or surgery as covariates.

► The study will focus on commonly used non-pharmacological interventions, such as CBT-I, acupuncture, yoga, Tai chi, Qigong, mindfulness therapy, physical exercise of different intensities (aerobic and resistance) , tuina and massage, which may lead to limitations to application of the findings for clinical guidance.

► We will only retrieve data from databases in Mandarin and English, which may restrict the amount of data available or lead to language bias.

PROSPERO registration number CRD42022382602.

INTRODUCTION

Insomnia, as one of the most prevalent complaints among cancer survivors, mainly

For peer review only - http://bmjopen.bmj.com/site/about/guidelines.xhtml

manifests as difficulty in initiating and/or maintaining sleep, early morning awakening, which are associated with impaired performance and daytime well being.[1] According to the epidemiological investigation, 36%-59% patients experienced insomnia during the cancer trajectories. Among them, rates were greater in patients with breast cancer from 42% to 69%.[2] In a 5-year follow-up investigation, 38% disease-free breast cancer survivors reported that they still had sleep problems.[3] Insomnia in breast cancer survivors can be debilitating, resulting in adverse impact on patients' life quality, and exacerbation on other symptoms, including pain, fatigue, hot flash and mental disorders.[4 5] Moreover, insomnia was associated with poor survival rate for patients with breast cancer.[6 7]

Currently the interventions in treating breast cancer related insomnia include pharmaceutical therapy and non-pharmaceutical ones. Due to the drug adverse effects, as well as the psychological and physical dependence, complementary and alternative medicine (CAM) has begun to receive more and more attention. According to the definition of CAM by NICCH, it is also called CIM (complementary and integrative medicine) or CIT (complementary integrative therapies), which includes four categories of treatment methods - natural nutrition supplements(such as dietary supplements, herbs and probiotics) , psychology (such as mindfulness, meditation)) , physical therapy (such as massage, spinal massage), a combination of psychosomatic exercises (such as yoga, tai chi, qigong, acupuncture) or psychological and nutritional (such as mindfulness diet) . [8] Cancer patients often use CAM therapy with recent estimates as high as 69%.[9] Most CAM methods, such as yoga, tai chi, meditation and music therapy, are safe for most patients. However, the use of herbs and supplements to treat sleep disturbance requires the supervision of health care professionals to prevent adverse reactions, interactions and toxicity.[10-12] Our network meta-analysis fully considered the compliance and safety of CAM in the treatment of insomnia in patients with breast cancer. Therefore, only non-drug intervention methods in CAM were retrieved, including psychotherapy (Cognitive-behavioral therapy for insomnia (CBT-I), mindfulness, psychological education guidance), psychosomatic exercise (yoga, tai chi, qigong, acupuncture) and different intensity physical exercise. The purpose is to

For peer review only - http://bmjopen.bmj.com/site/about/guidelines.xhtml

compare the differences and safety of non-pharmaceutical intervention in CAM in the treatment of insomnia in breast cancer, and offer some guidance and reference in clinical treatment.

Cognitive behavioral therapy for insomnia (CBT-I) has been demonstrated as an effective treatment and widely recommended for breast cancer survivors.[13 14] Evidence shows that acupuncture can improve sleep quality with mental and emotional distress symptoms.[15 16] Mind-body therapies including yoga, taichi or qigong also seem to be available for breast cancer related insomnia.[17 18] Besides, several studies reported that physical exercise can relieve the insomnia disorder of breast cancer patients.[19 20] Tuina, a non-pharmacological intervention based on TCM Zang-Fu organ and meridian theories, has also been proven to help improve insomnia [21]In conclusion, previous pairwise meta-analysis focused on only one CAM intervention mentioned above has demonstrated the efficacy in breast cancer related to insomnia.

However, apparent uncertainty about which non-pharmaceutical intervention is the most effective option makes it difficult for clinicians to manage this troublesome situation. In a randomized controlled trail, CBT-I showed superiority in shortening sleep onset latency, reducing wake, improving sleep efficiency while acupuncture was better at increasing total sleep time. Besides, the differences between acupuncture and CBT-I for women, non-whites, lower education, and those with pain were insignificant under subgroup analysis.[22] Thus, a comparison of these non-pharmaceutical interventions and the relative effectiveness between them represents an impending need for patients with breast cancer.

Network meta-analysis (NMA), which is also named multiple treatments comparison meta-analysis, helps to combine the direct and indirect evidence across a network of treatments. Furthermore, NMA is able to provide the ranking of treatment options based on their effectiveness.[23] However, to the best of our knowledge, so far NMA hasn't been used to perform the above functions in treating breast cancer related insomnia. Therefore, by the mean of systematic review (SR) and NMA, this study aims to compare the efficacy and evaluate the safety of different nonpharmaceutical CAM interventions including CBT-I, acupuncture, yoga, Tai chi, Qigong, mindfulness

For peer review only - http://bmjopen.bmj.com/site/about/guidelines.xhtml

therapy, physical exercise of different intensities (aerobic and resistance), tuina, and massage for breast cancer survivors with insomnia.

In previous studies, there was only one SR report on physical exercise and mind-body exercise in the treatment of insomnia in breast cancer, and there was no specific SR study on insomnia in breast cancer patients for other CAM therapies.[17] Based on our knowledge, this may be the first SR and NMA study to compare the efficacy and safety of non-pharmaceutical complementary and alternative therapies in breast cancer insomnia survivors according to ISI, PSQI, and sleep diary related measures. The results of the study will provide a ranking of different complementary alternative therapy interventions to assist patients, physicians, and policy makers in their decision making.

METHODS

Study registration

This protocol was registered on PROSPERO (CRD42022382602) and was reported following the Preferred Reporting Items for Systematic Review and Meta-Analysis Protocols (PRISMA-P) statement guidelines (online supplemental file 1 for PRISMA-P checklist). The findings of this study will be presented following the checklist of items to include when reporting a systematic review involving a network meta-analysis (PRISMA-NMA)

Inclusion criteria

Types of studies

We will incorporate RCTs that include multiple and single branches designs, full-text journal publications, unpublished clinical trials with online results in Chinese and English, and RCTs conducted in any healthcare setting.

Participants

Studies enrolled participants (18 years and older) who were diagnosed with breast cancer (stage 0-IV) and whose insomnia occurred after the breast cancer diagnosis. Patients who are receiving or have completed radiotherapy or chemotherapy or surgery

For peer review only - http://bmjopen.bmj.com/site/about/guidelines.xhtml

and patients with breast cancer who have never received radiotherapy or chemotherapy or surgery. There will be no restrictions on race, nationality, and education.

Types of interventions

Nonpharmaceutical CAM interventions, as the main treatment, will be included, which will be limited to several aspects of the following four treatments. (1)Psychological: CBT-I with at least 2 components of cognitive therapy, stimulus control, sleep hygiene (including sleep education), sleep restriction, relaxation training, and other variants of CBT-I; Mindfulness, Meditation； (2) Physical: massage, tuina, aerobic exercise, resistance exercise, walking, and interventions increasing physical activities; (3) Mind-body exercise (such as acupuncture, including acupuncture, electroacupuncture, auricular acupuncture, yoga, tai chi, Qigong). Studies used single or multiple complementary alternative medical intervention(s) will be considered.

Types of comparator(s)/control

The comparison group will be included usual care, a waiting control, placebo, sham acupuncture and sleep education.

Primary outcomes

The insomnia severity index (ISI) and the Pittsburgh sleep quality index (PSQI) will be considered as the primary outcomes in the study.

The ISI consists of 7 items to assess the nature and symptoms of the subject's sleep disorders. Questions address subjects' subjective ratings of sleep quality, including the severity of symptoms, subjects' satisfaction with their sleep patterns, the impact of the degree of insomnia on daily functioning, subjects' awareness of the impact of insomnia on themselves, and their level of frustration due to their sleep disorders.[24]

PSQI instrument produces a global sleep quality score and seven specific component scores: quality, latency, duration, disturbance, habitual sleep efficiency, use of sleeping medications, and daytime dysfunction. Global scores, ranging from 0 to 21 with higher scores, indicates poor sleep quality and high sleep disturbance.[25]

Secondary outcomes

Sleep efficiency, sleep latency (SOL), wake-up time after sleep onset (WASO), and total sleep time (TST) derived from polysomnography data, Actigraphy data or

For peer review only - http://bmjopen.bmj.com/site/about/guidelines.xhtml

sleep diaries will be regarded as secondary outcome measures. In addition, quantitative statistics of adverse events as another secondary indicator.[26]

Exclusion criteria

1. Studies that are duplicates.
2. Patients with other diseases that affect insomnia.
3. The design type is non- RCT, including conference paper, summary, abstracts, editorials, clinical observations, case studies, cohort studies, non-randomized trials, case-control studies, any non-experimental investigation (including cross-sectional and retrospective surveys).
4. We generally exclude studies involving various types of cancer unless they have subgroup data for breast cancer.
5. The full text of the study is not available.

Any study that meets one or more of the above criteria will not be considered.

Search methods for identification of studies

We will search all Chinese and English databases from the inception of the database to 31 December, 2022. The English literature databases include PubMed, Medline, Embase, Web of Science and Cochrane Central Register of Controlled Trials, and the Chinese literature databases include CBM, CNKI, VIP, WANFANG. The search keywords were focused on 'CAM' 'CIM', 'CIT', ' breast cancer ', ' insomnia ', ' acupuncture ', ' electroacupuncture ', ' Auricular acupuncture ', ' cognitive behavioral therapy ', ' Mind-body therapies ', 'mindfulness', 'meditation', 'tuina', 'masssage',' yoga ', ' tai chi ', ' qigong ', ' physical exercise ', ' resistance training ',' Walking ',' randomized controlled trial '. The final search formula was obtained by searching for the subject words, subordinate words and free words. The reference list of all selected articles will be independently screened to identify additional studies missed in the initial search. Table 1 shows specific search strategies for Web of science.

Table 1 Search strategy for the Web of Science

Number	Search items
**1**	TS=(breast neoplasms OR breast cancer OR breast tumor OR mammary cancer OR breast carcinoma)

For peer review only - http://bmjopen.bmj.com/site/about/guidelines.xhtml

**2**	TS=(sleep initiation and maintenance disorders OR insomnia)
**3**	TS=(complementary therapies OR complementary medicine OR alternative medicine OR alternative therapies OR integrative medicine
**4**	TS=(acupuncture OR electroacupuncture OR auricular acupuncture OR tuina OR massage)
**5**	TS=(cognitive behavioral therapy OR mindfulness OR meditation)
**6**	TS=(mind-body therapies)
**7**	TS=(yoga)
**8**	TS=(tai ji OR tai chi)
**9**	TS=(qigong)
**10**	TS=(exercise therapy OR physical exercise OR aerobic exercise)
**11**	TS=(resistance training)
**12**	TS=(walking)
**13**	TS=(randomized controlled trial OR controlled clinical trial OR randomized OR placebo OR clinical trials as topic OR randomly OR trial)
**14**	TS=(animals)
**15**	TS=(humans AND animals)
**16**	((#13) NOT #14) NOT #15
**17**	#3 OR #4 OR #5 OR #6 OR #7 OR #8 OR #9 OR #10 OR #11 OR #12
**18**	#1 AND #2 AND #16 AND #17

Data collection and analysis

Selection of studies

Two researchers (QJ and LML) will screen the literature independently and extract the data and cross-check them. If there are differences, these will be resolved through discussion or negotiation with third parties. In the literature screening, the title of the article will be first read. After excluding obviously unrelated literatures, the abstract and full text will be further read to determine whether it will be included. If necessary, the authors of the original studies will be contacted by email or telephone to obtain information that has not been identified but is important to this study. EndnoteX9 will be used to manage retrieved studies and remove duplicates. The selection procedure is presented in a PRISMA flow chart (Figure 1).

Data extraction and management

Two researchers (QJ and LML) will independently extract data based on a predesigned form. The extracted data will be as follows:(1) Basic information included in the study: first author and year of publication; (2) Research methods: research type,

For peer review only - http://bmjopen.bmj.com/site/about/guidelines.xhtml

intervention measures, control measures, sample size; (3) Concerned Outcome indicators and outcome measurement data. (4) Follow-up. The corresponding authors will be contacted for missing information. The two researchers will crosscheck the data after completion of data extraction. The disagreements will be solved by the team discussion or consultation with the third researcher (YLC).

Quality assessment

Two independent reviewers (QJ and PY) will complete the risk-of-bias assessment and evaluation of evidence certainty.

We will use Version 2 of the Cochrane tool for assessing the risk of bias in randomized trials (RoB2) revised by the Cochrane Methodology Group in 2019.[27] The quality of the study will be evaluated in the following five areas: (1) bias in the randomization process, (2) deviation from established interventions, (3) bias in missing outcome data, (4) bias in outcome measurement, and (5) selective reporting of bias in results.

The Grading of Recommendations Assessment, Development and Evaluation System (GRADE) system will be used to grade the quality of the evidence for main outcomes.[28] Evidence quality will be rated ‘high’, ‘moderate’, ‘low’ or ‘very low’ according to the GRADE rating standards. The evidence quality of a specific study will be assessed according to the risk of bias, inconsistency, indirectness, imprecision and publication bias.

Pairwise meta-analysis

The pairwise meta-analysis will be performed using STATA 15.0. Conventional pairwise meta-analyses will be performed with random-effects model. Pooled odds ratio (OR) with 95% confidence interval (CI) will be used for the dichotomous variables. Standardized mean differences (SMDs) or weighted mean differences (WMDs) with 95% CI will be used for the continuous variables. The heterogeneity between trials will be estimated using I^2 statistics and P value. The values of 25%, 50%, and 75% for the I^2 as indicative of low, moderate, and high statistical heterogeneity, respectively. We will explore sources of heterogeneity by subgroup analysis and meta-regression.[29]

Network meta-analysis

For peer review only - http://bmjopen.bmj.com/site/about/guidelines.xhtml

If enough data is available, we will conduct a network meta-analysis (NMA) to compare the efficacy of different interventions and controls. The NMA will be conducted on both direct evidence and indirect evidence in a Bayesian framework using STATA V.15.0 software. The publication bias will be evaluated with the Egger's test and funnel plots if the number of studies exceeds. A 2-tailed P value < 0.05 is considered as statistically significant.[30] In addition, a node-splitting model will also be used to assess local inconsistency at the network level, and a consistency or inconsistency model will be selected based on the results. If $P<0.05$, there was a statistically significant difference between the indirect and direct multiple treatment comparisons. To evaluate the convergence of the results, we will analyze the potential scale reduction factor (PSRF), where a PSRF value close to 1 indicates successful convergence.[31]

Subgroup analysis, meta regression analysis and sensitivity analysis

Subgroup analysis and meta-regression analysis will be used to explore possible sources of heterogeneity and inconsistency. Subgroup analysis will be performed according to the different types of non-pharmaceutical interventions. We will conduct a meta-regression analysis with participants who have received or are receiving or have never received chemotherapy, radiation, or surgery as covariates. Sensitivity analysis is carried out by the method of exclusion to verify the robustness of the results.

Publication bias assessment

If more than 10 studies are included, a comparison-adjusted funnel plot will be generated to detect reporting bias.

Patients and public involvement

This is a SR and NMA based on existing studies, so no patients or members of the public will directly be involved.

Ethics and dissemination

The study will not require ethical approval because it comprises analysis based on existing studies. The results are expected to be published in a peer-reviewed journal or disseminated at relevant conferences. The objective of our study will evaluate the

For peer review only - http://bmjopen.bmj.com/site/about/guidelines.xhtml

impact of complementary alternative therapies (including acupuncture, CBT-I, physical exercise, mind-body exercise etc.) on sleep-related outcomes in breast cancer survivors with insomnia by conducting a systematic review and network meta-analysis of existing randomized controlled trial data for the purpose of providing reliable evidence-based medical evidence for complementary and alternative therapies for insomnia in breast cancer.

Contributors

QJ and LML contributed equally to this article as co-first authors. YLC and PY contributed to the design of the study. The manuscript was drafted by QJ and LML. QJ and LML participated in the design of the search strategy and data extraction dataset. YLC and PY will monitor each procedure of the review and be responsible for quality control. All the authors participated in the reading, discussion and revision of the manuscript, and all approved of the protocol for publication.

Funding

This work was supported by the Clinical Incubation Program of the National Medical Center of LongHua Hospital to Shanghai University of Traditional Chinese Medicine, with grant number (GY202201).

Competing interests

None declared.

Patient and public involvement

Patients and/or the public were not involved in the design, conduct, reporting, or dissemination plans of this research.

Patient consent for publication

Not applicable.

Provenance and peer review

Not commissioned; externally peer reviewed.

Reference

1. Schieber K, Niecke A, Geiser F, et al. The course of cancer-related insomnia: don't expect it to disappear after cancer treatment. *Sleep medicine* 2019;**58**:107-13.

For peer review only - http://bmjopen.bmj.com/site/about/guidelines.xhtml

2. Savard J, Ivers H, Villa J, et al. Natural course of insomnia comorbid with cancer: an 18-month longitudinal study. *Journal of clinical oncology : official journal of the American Society of Clinical Oncology* 2011;**29**(26):3580-6.

3. Schmidt ME, Wiskemann J, Steindorf K. Quality of life, problems, and needs of disease-free breast cancer survivors 5 years after diagnosis. *Quality of life research : an international journal of quality of life aspects of treatment, care and rehabilitation* 2018;**27**(8):2077-86.

4. Collins KP, Geller DA, Antoni M, et al. Sleep duration is associated with survival in advanced cancer patients. *Sleep medicine* 2017;**32**:208-12.

5. Narayanan S, Reddy A, Lopez G, et al. Sleep disturbance in cancer patients referred to an ambulatory integrative oncology consultation. *Supportive care in cancer : official journal of the Multinational Association of Supportive Care in Cancer* 2022;**30**(3):2417-25.

6. Costa AR, Fontes F, Pereira S, et al. Impact of breast cancer treatments on sleep disturbances - A systematic review. *Breast (Edinburgh, Scotland)* 2014;**23**(6):697-709.

7. Savard J, Ivers H, Savard MH, et al. Cancer treatments and their side effects are associated with aggravation of insomnia: Results of a longitudinal study. *Cancer* 2015;**121**(10):1703-11.

8. National Center for Complementary and Integrative Health (NCCIH) (2016) Complementary, alternative, or integrative health: what's in a name? Secondary National Center for Complementary and Integrative Health (NCCIH) (2016) Complementary, alternative, or integrative health: what's in a name? https://nccih.nih.gov/health/integrative-health#integrative.

9. Smith JA. Assessment of the Utilization of Complimentary and Alternative Medication in Women with Gynecologic or Breast Cancers. 2015.

10. White CM. Dietary Supplements Pose Real Dangers to Patients. *The Annals of pharmacotherapy* 2020;**54**(8):815-19.

11. Shinjyo N, Waddell G, Green J. Valerian Root in Treating Sleep Problems and Associated Disorders-A Systematic Review and Meta-Analysis. *Journal of evidence-based integrative medicine* 2020;**25**:2515690x20967323.

12. Richardson MA, Sanders T, Palmer JL, et al. Complementary/alternative medicine use in a comprehensive cancer center and the implications for oncology. *Journal of clinical oncology : official journal of the American Society of Clinical Oncology* 2000;**18**(13):2505-14.

13. Ma Y, Hall DL, Ngo LH, et al. Efficacy of cognitive behavioral therapy for insomnia in breast cancer: A meta-analysis. *Sleep medicine reviews* 2021;**55**:101376.

14. Bean HR, Diggens J, Ftanou M, et al. Light enhanced cognitive behavioral therapy for insomnia and fatigue during chemotherapy for breast cancer: a randomized controlled trial. *Sleep* 2022;**45**(3).

15. Zhang J, Zhang Z, Huang S, et al. Acupuncture for cancer-related insomnia: A systematic review and meta-analysis. *Phytomedicine : international journal of phytotherapy and phytopharmacology* 2022;**102**:154160.

16. Garland SN, Xie SX, Li Q, et al. Comparative effectiveness of electro-acupuncture versus gabapentin for sleep disturbances in breast cancer survivors with hot flashes: a randomized trial. *Menopause (New York, NY)* 2017;**24**(5):517-23.

17. Kreutz C, Schmidt ME, Steindorf K. Effects of physical and mind-body exercise on sleep problems during and after breast cancer treatment: a systematic review and meta-analysis. *Breast cancer research and treatment* 2019;**176**(1):1-15.

For peer review only - http://bmjopen.bmj.com/site/about/guidelines.xhtml

18. Chaoul A, Milbury K, Spelman A, et al. Randomized trial of Tibetan yoga in patients with breast cancer undergoing chemotherapy. *Cancer* 2018;**124**(1):36-45.

19. Mercier J, Savard J, Bernard P. Exercise interventions to improve sleep in cancer patients: A systematic review and meta-analysis. *Sleep medicine reviews* 2017;**36**:43-56.

20. Steindorf K, Wiskemann J, Ulrich CM, et al. Effects of exercise on sleep problems in breast cancer patients receiving radiotherapy: a randomized clinical trial. *Breast cancer research and treatment* 2017;**162**(3):489-99.

21. Wang Z, Xu H, Zhou H, et al. A systematic review with meta-analysis: Traditional Chinese tuina therapy for insomnia. *Frontiers in neuroscience* 2022;**16**:1096003.

22. Garland SN, Xie SX, DuHamel K, et al. Acupuncture Versus Cognitive Behavioral Therapy for Insomnia in Cancer Survivors: A Randomized Clinical Trial. *Journal of the National Cancer Institute* 2019;**111**(12):1323-31.

23. Bafeta A, Trinquart L, Seror R, et al. Reporting of results from network meta-analyses: methodological systematic review. *BMJ (Clinical research ed)* 2014;**348**:g1741.

24. Morin CM, Belleville G, Bélanger L, et al. The Insomnia Severity Index: psychometric indicators to detect insomnia cases and evaluate treatment response. *Sleep* 2011;**34**(5):601-8.

25. Buysse DJ, Reynolds CF, 3rd, Monk TH, et al. The Pittsburgh Sleep Quality Index: a new instrument for psychiatric practice and research. *Psychiatry research* 1989;**28**(2):193-213.

26. Fekedulegn D, Andrew ME, Shi M, et al. Actigraphy-Based Assessment of Sleep Parameters. *Annals of work exposures and health* 2020;**64**(4):350-67.

27. Sterne JAC, Savović J, Page MJ, et al. RoB 2: a revised tool for assessing risk of bias in randomised trials. *BMJ (Clinical research ed)* 2019;**366**:l4898.

28. Guyatt GH, Oxman AD, Vist GE, et al. GRADE: an emerging consensus on rating quality of evidence and strength of recommendations. *BMJ (Clinical research ed)* 2008;**336**(7650):924-6.

29. Higgins JP, Thompson SG, Deeks JJ, et al. Measuring inconsistency in meta-analyses. *BMJ (Clinical research ed)* 2003;**327**(7414):557-60.

30. Salanti G, Ades AE, Ioannidis JP. Graphical methods and numerical summaries for presenting results from multiple-treatment meta-analysis: an overview and tutorial. *Journal of clinical epidemiology* 2011;**64**(2):163-71.

31. BROOKS, Stephen P, GELMAN, et al. General methods for monitoring convergence of iterative simulations. 1998.

Figure Legend

Figure 1 PRISMA flow diagram of the study selection process

Table

Table 1 Search strategy for the Web of Science

For peer review only - http://bmjopen.bmj.com/site/about/guidelines.xhtml

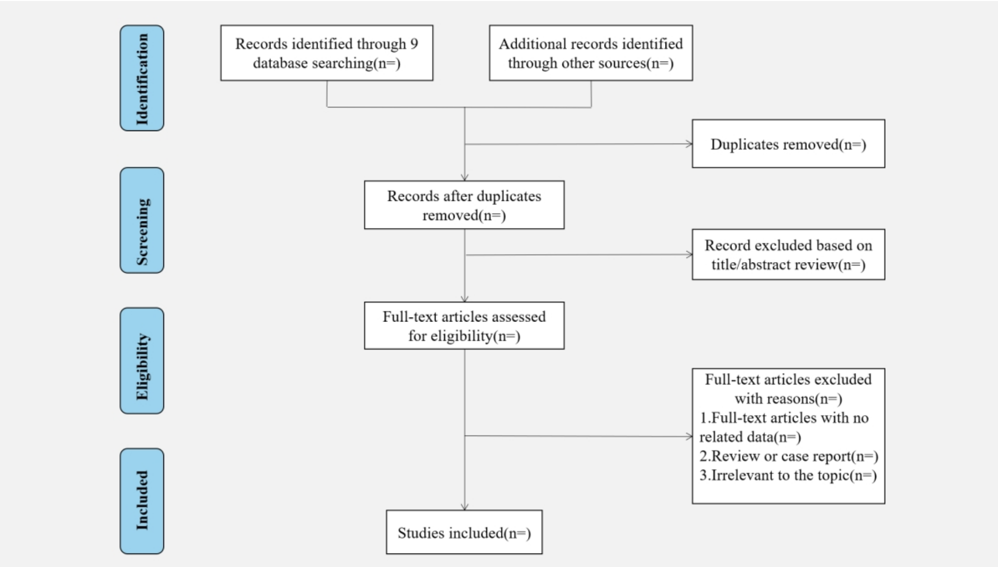

Figure 1 PRISMA flow diagram of the study selection process

276x167mm (192 x 192 DPI)

Search strategies

Pubmed:

(((("breast neoplasms"[Mesh]) OR (breast cancer[tiab]) OR (breast tumor[tiab]) OR (mammary cancer[tiab]) OR (breast carcinoma[tiab])) AND (("sleep initiation and maintenance disorders"[Mesh]) OR (insomnia[tiab]))) AND ((("complementary therapies"[Mesh]) OR (complementary medicine[tiab]) OR (alternative medicine[tiab]) OR (alternative therapies[tiab]) OR (integrative medicine[tiab])) OR (("acupuncture"[Mesh]) OR (electroacupuncture[tiab]) OR (auricular acupuncture[tiab])) OR ("cognitive behavioral therapy"[Mesh]) OR ("massage"[Mesh]) OR (tuina[tiab]) OR ("mindfulness"[Mesh]) OR ("meditation"[Mesh]) OR ("mind-body therapies"[Mesh]) OR ("yoga"[Mesh]) OR (("tai ji"[Mesh]) OR (tai chi[tiab])) OR ("qigong"[Mesh]) OR (("exercise"[Mesh]) OR (physical exercise[tiab]) OR (aerobic exercise[tiab])) OR ("resistance training"[Mesh]) OR ("walking"[Mesh]))) AND ((randomized controlled trial[pt] OR controlled clinical trial[pt] OR randomized[tiab] OR placebo[tiab] OR randomly[tiab] OR trial[tiab] OR groups[tiab]) NOT (animals[mh] NOT humans[mh]))

Web of science:

Table 1 Search strategy for the Web of Science

Number	Search items
1	TS=(breast neoplasms OR breast cancer OR breast tumor OR mammary cancer OR breast carcinoma)
2	TS=(sleep initiation and maintenance disorders OR insomnia)
3	TS=(complementary therapies OR complementary medicine OR alternative medicine OR alternative therapies OR integrative medicine
4	TS=(acupuncture OR electroacupuncture OR auricular acupuncture OR tuina OR massage)
5	TS=(cognitive behavioral therapy OR mindfulness OR meditation)
6	TS=(mind-body therapies)
7	TS=(yoga)

For peer review

**8**	TS=(tai ji OR tai chi)
**9**	TS=(qigong)
**10**	TS=(exercise therapy OR physical exercise OR aerobic exercise)
**11**	TS=(resistance training)
**12**	TS=(walking)
**13**	TS=(randomized controlled trial OR controlled clinical trial OR randomized OR placebo OR clinical trials as topic OR randomly OR trial)
**14**	TS=(animals)
**15**	TS=(humans AND animals)
**16**	((#13) NOT #14) NOT #15
**17**	#3 OR #4 OR #5 OR #6 OR #7 OR #8 OR #9 OR #10 OR #11 OR #12
**18**	#1 AND #2 AND #16 AND #17

Medline:

1. breast neoplasms[Mesh] or (breast cancer or breast tumor or mammary cancer or breast carcinoma).ti,ab.

2. (sleep initiation and maintenance disorders).mp. or (insomnia).ti,ab.

3. complementary therapies[Mesh] or (complementary medicine or alternative medicine or alternative therapies or integrative medicine).ti,ab.

4. acupuncture[Mesh] or (electroacupuncture or auricular acupuncture).ti,ab.

5. massage[Mesh] or (tuina).ti,ab.

6. cognitive behavioral therapy[Mesh]

7. mindfulness[Mesh]

8. meditation[Mesh]

9. mind-body therapies[Mesh]

10. yoga[Mesh]

11. tai ji[Mesh] or (tai chi).ti,ab.

12. qigong[Mesh]

13. exercise[Mesh] or (physical exercise or aerobic exercise).ti,ab.

14. resistance training[Mesh]

For peer review only

15. walking[Mesh]

16. (randomized controlled trial or controlled clinical trial).pt. or random$.ab. or trial.ab.

17. 3 or 4 or 5 or 6 or 7 or 8 or 9 or 10 or 11 or 12 or 13 or 14 or 15

18. 1 and 2 and 16 and 17

Embase:

1. 'breast neoplasms'/exp OR 'breast neoplasms' OR 'breast cancer':ab,ti OR 'breast tumor':ab,ti OR 'mammary cancer':ab,ti OR 'breast carcinoma':ab,ti

2. 'sleep initiation and maintenance disorder'/exp OR 'sleep initiation and maintenance disorder' OR 'insomnia':ab,ti

3. 'complementary therapies'/exp OR 'complementary therapies' OR 'complementary medicine':ab,ti OR 'alternative medicine':ab,ti OR 'alternative therapies':ab,ti OR 'integrative medicine':ab,ti

4. 'acupuncture'/exp OR 'acupuncture' OR 'electroacupuncture':ab,ti OR 'auricular acupuncture':ab,ti

5. 'massage'/exp OR 'massage' OR 'tuina':ab,ti

6. 'cognitive behavioral therapy'/exp OR 'cognitive behavioral therapy'

7. 'mindfulness'/exp OR 'mindfulness'

8. 'meditation'/exp OR 'meditation'

9. 'mind-body therapies'/exp OR 'mind-body therapies'

10. 'yoga'/exp OR 'yoga'

11. 'tai ji'/exp OR 'tai ji' OR 'tai chi':ab,ti

12. 'qigong'/exp OR 'qigong'

13. 'exercise'/exp OR 'exercise' OR 'physical exercise':ab,ti OR 'aerobic exercise':ab,ti

14. 'resistance training'/exp OR 'resistance training'

15. 'walking'/exp OR 'walking'

16. 'crossover procedure':de OR 'double-blind procedure':de OR 'randomized controlled trial':de OR 'single-blind procedure':de OR (random* OR factorial* OR crossover* OR cross NEXT/1 over* OR placebo* OR doubl* NEAR/1 blind* OR singl* NEAR/1 blind* OR assign* OR allocat* OR volunteer*):de,ab,ti

For peer review only

17. 3 or 4 or 5 or 6 or 7 or 8 or 9 or 10 or 11 or 12 or 13 or 14 or 15

18. 1 and 2 and 16 and 17

Cochrane:

#1 ((breast neoplasms) OR (breast cancer) OR (breast tumor) OR (mammary cancer) OR (breast carcinoma)):ab,ti,kw

#2 (sleep initiation and maintenance disorders) OR (insomnia):ab,ti,kw

#3 (((complementary therapies) OR (complementary medicine) OR (alternative medicine) OR (alternative therapies) OR (integrative medicine)) OR ((acupuncture) OR (electroacupuncture) OR (auricular acupuncture) OR (tuina) OR (massage)) OR ((cognitive behavioral therapy) OR (mindfulness) OR (meditation)) OR (mind-body therapies) OR (yoga) OR ((tai ji) OR (tai chi)) OR (qigong) OR ((exercise therapy) OR (physical exercise) OR (aerobic exercise)) OR (resistance training) OR (walking)):ab,ti,kw

#1 AND #2 AND #3

CBM:

("失眠"[摘要] OR "不寐"[摘要] OR "睡眠障碍"[摘要] OR "睡眠"[摘要]) AND ("乳腺癌"[摘要] OR "乳腺肿瘤"[摘要]) AND ("补充医学"[摘要] OR "替代医学"[摘要] OR "结合医学"[摘要] OR "针刺"[摘要] OR "针灸"[摘要] OR "电针"[摘要] OR "耳针"[摘要] OR "推拿"[摘要] OR "按摩"[摘要] OR "正念"[摘要] OR "冥想"[摘要] OR "认知行为"[摘要] OR "瑜伽"[摘要] OR "太极"[摘要] OR "气功"[摘要] OR "有氧"[摘要] OR "运动"[摘要] OR "体育锻炼"[摘要] OR "身心"[摘要] OR "抗阻"[摘要])

CNKI:

(TKA = '失眠' OR TKA = '不寐' OR TKA = '睡眠' OR TKA = '睡眠障碍') AND (TKA = '乳腺癌' OR TKA = '乳腺肿瘤') AND (TKA = '补充医学' OR TKA = '替代医学' OR TKA = '结合医学' OR TKA = '针刺' OR TKA = '针灸' OR TKA = '电针' OR TKA = '耳针' OR TKA = '推拿' OR TKA = '按摩' OR TKA = '正念' OR TKA = '冥想' OR TKA = '认知行为' OR TKA = '瑜伽' OR TKA = '太极' OR TKA = '气功'

For peer review only - http://bmjopen.bmj.com/site/about/guidelines.xhtml

OR TKA = '有氧' OR TKA = '运动' OR TKA = '体育锻炼' OR TKA = '身心' OR TKA = '抗阻')

VIP：

M=(失眠 OR 不寐 OR 睡眠 OR 睡眠障碍) AND M=(乳腺癌 OR 乳腺肿瘤) AND M=(补充医学 OR 替代医学 OR 结合医学 OR 针刺 OR 针灸 OR 电针 OR 耳针 OR 推拿 OR 按摩 OR 正念 OR 冥想 OR 认知行为 OR 瑜伽 OR 太极 OR 气功 OR 有氧 OR 运动 OR 体育锻炼 OR 身心 OR 抗阻)

WANFANG：

题名或关键词:(("失眠" or "不寐" or "睡眠" or "睡眠障碍") and ("乳腺癌" or "乳腺肿瘤") and ("补充医学" or "替代医学" or "结合医学" or "针刺" or "针灸" or "电针" or "耳针" or "推拿" or "按摩" or "正念" or "冥想" or "认知行为" or "瑜伽" or "太极" or "气功" or "有氧" or "运动" or "体育锻炼" or "身心" or "抗阻"))

For peer review only

PRISMA-P (Preferred Reporting Items for Systematic review and Meta-Analysis Protocols) 2015 checklist: recommended items to address in a systematic review protocol*

Section and topic	Item No	Checklist item	Location in the article
**ADMINISTRATIVE INFORMATION**			
Title:			
Identification	1a	Identify the report as a protocol of a systematic review	Page 1, Lines 2-3
Update	1b	If the protocol is for an update of a previous systematic review, identify as such	NA
Registration	2	If registered, provide the name of the registry (such as PROSPERO) and registration number	Page 4 , Line 28
Authors:			
Contact	3a	Provide name, institutional affiliation, e-mail address of all protocol authors; provide physical mailing address of corresponding author	Page 1 , Lines 6-29
Contributions	3b	Describe contributions of protocol authors and identify the guarantor of the review	Page 11 , Lines 29-30<br>Page 12 , Lines 1-5
Amendments	4	If the protocol represents an amendment of a previously completed or published protocol, identify as such and list changes; otherwise, state plan for documenting important protocol amendments	NA
Support:			
Sources	5a	Indicate sources of financial or other support for the review	Page 12 , Lines 6-9
Sponsor	5b	Provide name for the review funder and/or sponsor	Page 12 , Lines 6-9
Role of sponsor or funder	5c	Describe roles of funder(s), sponsor(s), and/or institution(s), if any, in developing the protocol	NA
**INTRODUCTION**			
Rationale	6	Describe the rationale for the review in the context of what is already known	Page 3 , Lines 12-30<br>Page 4 , Lines 1-21
Objectives	7	Provide an explicit statement of the question(s) the review will address with reference to participants, interventions, comparators, and outcomes (PICO)	Page 4 , Lines 22-24
**METHODS**			
Eligibility criteria	8	Specify the study characteristics (such as PICO, study design, setting, time frame) and report characteristics (such as years considered, language, publication status) to be used as criteria for eligibility for the review	Page 5 , Lines 5-29<br>Page 6 , Lines 1-28
Information sources	9	Describe all intended information sources (such as electronic databases, contact with study authors, trial registers or other grey literature sources) with planned dates of coverage	Page 7 , Lines 1-12

For peer review only

Search strategy	10	Present draft of search strategy to be used for at least one electronic database, including planned limits, such that it could be repeated	Page 7 , Table1
Study records:			
Data management	11a	Describe the mechanism(s) that will be used to manage records and data throughout the review	Page 8 , Line 10
Selection process	11b	State the process that will be used for selecting studies (such as two independent reviewers) through each phase of the review (that is, screening, eligibility and inclusion in meta-analysis)	Page 8 , Lines 3-9
Data collection process	11c	Describe planned method of extracting data from reports (such as piloting forms, done independently, in duplicate), any processes for obtaining and confirming data from investigators	Page 8 , Lines 18-20
Data items	12	List and define all variables for which data will be sought (such as PICO items, funding sources), any pre-planned data assumptions and simplifications	Page 8 , Lines 14-17
Outcomes and prioritization	13	List and define all outcomes for which data will be sought, including prioritization of main and additional outcomes, with rationale	Page 6 , Lines 1-17
Risk of bias in individual studies	14	Describe anticipated methods for assessing risk of bias of individual studies, including whether this will be done at the outcome or study level, or both; state how this information will be used in data synthesis	Page 9 , Lines 1-2
Data synthesis	15a	Describe criteria under which study data will be quantitatively synthesised	Page 9 , Lines 13-30<br>Page 10 , Lines 1-5
	15b	If data are appropriate for quantitative synthesis, describe planned summary measures, methods of handling data and methods of combining data from studies, including any planned exploration of consistency (such as I^2, Kendall's τ)	Page 9 , Lines 13-22
	15c	Describe any proposed additional analyses (such as sensitivity or subgroup analyses, meta-regression)	Page 10 , Lines 6-13
	15d	If quantitative synthesis is not appropriate, describe the type of summary planned	Page 9 , Lines 13-30<br>Page 10 , Lines 1-5
Meta-bias(es)	16	Specify any planned assessment of meta-bias(es) (such as publication bias across studies, selective reporting within studies)	Page 10 , Lines 14-16
Confidence in cumulative evidence	17	Describe how the strength of the body of evidence will be assessed (such as GRADE)	Page 9 , Lines 7-12

*** It is strongly recommended that this checklist be read in conjunction with the PRISMA-P Explanation and Elaboration (cite when available) for important clarification on the items. Amendments to a review protocol should be tracked and dated. The copyright for PRISMA-P (including checklist) is held by the PRISMA-P Group and is distributed under a Creative Commons Attribution Licence 4.0.**

From: Shamseer L, Moher D, Clarke M, Ghersi D, Liberati A, Petticrew M, Shekelle P, Stewart L, PRISMA-P Group. Preferred reporting items for systematic review and meta-analysis protocols (PRISMA-P) 2015: elaboration and explanation. BMJ. 2015 Jan 2;349(jan02 1):g7647.

For peer review only
